# One year quality of life outcomes in critically ill children: a multicenter prospective cohort study

**DOI:** 10.1186/s13054-026-06084-9

**Published:** 2026-05-16

**Authors:** Joseph C. Manning, Jos M. Latour, Elizabeth Draper, Philip Quinlan, Emma M. Popejoy, Grazziela Figueredo, Shreya Iyer, Thomas Trimble, Julie Menzies, Martha A. Q. Curley, Jane Coad, The OCEANIC Study Investigators, Eugenia Abaleke, Hawakin Haji Ali, Kathryn Allison, Laura Anderson, Taya Anderson, Lydia Ashton, Katherine Baptiste, Holly Belfield, Sarah Benkenstein, Lara Bunni, Amy Burrow, Ushra Chandran, Charlene Davis, Laura Dodge, Rachael Dore, Sarah Fox, Ritika Ghosh-Dastidar, Samantha Glover, Sarah Goodwin, Georgina Harlow, Paris-Lucia Harrison, Ellen Haskins, Tara McHugh, Katie Ireland, Madiha Islam, Navjyot Jabbal, Claire Jennings, Dawn Jones, Nosheen Khalid, Neelam Khan, Tahmina Khatun, Klaudia Kupiec, Becca Lean, Annabel Little, Rachel Loughead, Hannah Malkin, Ilham Manjra, Rebecca Marshall, Shelley Mayor, Hamza Meghari, Francesca Moody, Rachael Morrison, Chengeto Muhaso, Helen Marley Munn, Lucy Murphy, Lauran O’Neill, Samantha Owen, Laura O’Malley, Harriet Payne, Caroline Payne, Joana Gomes De Queiroz, Laura Rad, Natalie Read, Ceri Robbins, Kevin Samuels, Ramesh Sathyamurthy, Gemma Sedgwick, Theresa Simangan, Elizabeth Stoddart, Jivika Talwar, Ana Louisa Thomas, Carly Tooke, Natalie Turner, Helen Vander-Johnson, Roxanne Williams, Helen Winmill

**Affiliations:** 1 School of Healthcare, University of Leicester, Leicester, UK; 2 Nottingham Children’s Hospital, Nottingham University Hospitals NHS Trust, Nottingham, UK; 3 School of Nursing and Midwifery, University of Plymouth, Plymouth, UK; 4 Nursing Department, Zhongshan Hospital of Fudan University, Shanghai, China; 5 Curtin School of Nursing, Curtin University, Perth, Australia; 6 Department of Health Sciences, University of Leicester, Leicester, Leicestershire UK; 7 Faculty of Medicine & Health Sciences, University of Nottingham, Nottingham, UK; 8 Nottingham Children’s Hospital, Nottingham University Hospitals NHS Trust, Nottingham, UK; 9 School of Health Sciences, University of Nottingham, Nottingham, UK; 10 Advanced Data Analysis Centre, Faculty of Medicine & Health Sciences, University of Nottingham, Nottingham, UK; 11 Paediatric Intensive Care Unit, Bristol Royal Hospital for Children, University Hospitals Bristol and Weston NHS Foundation Trust, Bristol, UK; 12 Department of Family and Community Health, School of Nursing, University of Pennsylvania, Philadelphia, PA USA; 13 Anesthesia and Critical Care Medicine, Perelman School of Medicine, University of Pennsylvania, Philadelphia, PA USA; 14 The Research Institute, Children’s Hospital of Philadelphia, Philadelphia, PA USA; 15 University Hospital Coventry and Warwickshire NHS Foundation Trust, Coventry, UK

**Keywords:** Pediatric intensive care, Long-term outcomes, Health‑related quality of life, Recovery trajectories, PedsQL, Machine learning

## Abstract

**Background:**

Survivors of pediatric intensive care often experience prolonged morbidity, but recovery trajectories and features associated with impairment in general PICU populations remain uncertain. We aimed to explore the trajectory of health-related quality of life (HRQoL) and fatigue in critically ill children over the first year following PICU discharge, and to identify baseline and PICU factors associated with worse outcomes.

**Methods:**

OCEANIC is a multicenter prospective cohort study across 10 English PICUs. Children aged 1 month–17 years with PICU stay ≥ 48 h were enrolled (2019–2022) and followed for 12-months (to 2023). HRQoL (PedsQL™ 4.0 Acute Versions) and fatigue (PedsQL™ Multidimensional Fatigue) were assessed at baseline (pre‑admission), PICU discharge, and 1, 3, 6 and 12 -months. We used Random Forest models with SHapley Additive exPlanations (SHAP) to identify features associated with below‑baseline HRQoL at each timepoint.

**Results:**

Of 326 children enrolled, 220 had ≥ 3 HRQoL assessments. Mean PedsQL fell from 73.3 (SD 20.99) at baseline to 54.3 (SD 23.52) at discharge, then rose to 62.9 (1-month, SD 21.73) and 67.1 (3-months, SD 20.38), stabilizing thereafter (70.4 (SD 21.34) at 6-months; 69.8 (SD 22.46) at 12-months; p < 0.001 across time). At discharge, 71.8% were below their baseline HRQoL. Among 12‑month respondents, 58.1% remained below their baseline. Physical and school functioning showed persistent impairment, with cognitive functioning returning to baseline by 1-month. Fatigue largely normalized by 6-months. Higher baseline HRQoL and older age were consistently influential features with worse HRQoL, with physiological/illness markers important features across timepoints.

**Conclusion:**

At 12-months, 58% of responding children remained below their pre-PICU baseline HRQoL, with persistent impairment most evident in physical and school functioning. Modelling identifies population-level subgroups of children characterized by higher baseline or lower discharge HRQoL, older age, and prolonged PICU exposure who may warrant closer multidisciplinary follow‑up after PICU discharge.

**Trial registration:**

ISRCTN28072812 14/02/2020

**Supplementary Information:**

The online version contains supplementary material available at 10.1186/s13054-026-06084-9.

## Background

Survival of the paediatric intensive care unit (PICU) has improved, shifting attention from mortality to longer‑term outcomes [1]. Cohort and qualitative studies indicate that survivors experience deficits in health-related quality of life (HRQoL) [2], with psychosocial and transitional impact on children and their families [3, 4]. However, most studies focus on specific diagnoses, single time points, or lack pre‑PICU status, limiting inference about recovery trajectories in general PICU populations [5].

Few studies account for pre-PICU status, hindering comprehensive understanding of recovery trajectories and long-term outcomes in the general PICU population. The Post Intensive Care Syndrome in pediatrics (PICS-p) framework offers a conceptual basis for understanding these long-term effects [6], but the evidence-base, trajectory, and associated risk factors are still poorly defined.

Given the complexity of recovery after paediatric critical illness, there is increasing interest in analytic approaches that can integrate multiple clinical and contextual variables. Explainable machine‑learning methods offer a means of identifying patterns within such high‑dimensional data. However, these approaches describe associations within fitted models rather than causal relationships and must be interpreted cautiously within observational clinical datasets.

### Methods

We aimed to characterize trajectories of HRQoL and fatigue over the first year after PICU discharge in a multicenter cohort, and to identify baseline and PICU factors associated with below-baseline HRQoL at 1-, 3-, 6- and 12-months. OCEANIC (Outcomes of ChildrEn and fAmilies after paediatric INtensive Care) is a multicenter prospective cohort study across 10 English PICUs.. Enrolment took place from November 2019 to May 2022, with follow‑up completed in May 2023. The study is reported as per STROBE guidelines for observational studies [7].

The study methods have been published previously by Manning et al., [8]. Study modifications were implemented in response to the COVID-19 pandemic [9] and emerging evidence including: (i) increase in sample size from 300 to 334 to account for attrition; (ii) extending enrolment from 12- to 28-months due to national research pauses; (iii) expanding sites from 5 to 10; and (iv) reducing the minimum PICU length-of-stay from 72 to 48 h.

We included critically ill children: (a) aged 1-month to 17-years and discharged from the PICU alive, (b) had a PICU total length-of-stay ≥ 48 h and who received ICU therapies, (c) living with at least one parent/legal guardian. Exclusion criteria were: (a) had a previous Neonatal/PICU admission, (b) had an active end-of-life pathway or (c) had ongoing safeguarding procedures.

### Data collection

Clinical and demographic data were extracted from the Paediatric Intensive Care Audit Network (PICANet) dataset, the national audit database for paediatric intensive care in the United Kingdom [10]. Follow-up data were prospectively collected at PICU discharge and at 1-, 3-, 6-, and 12-months post-discharge. Baseline data, collected at enrolment, reflected the child’s state 2-weeks prior to PICU admission. Data collection methods (face-to-face, telephone, online, or mail) were based on family preference. Children (aged > 6 years) self-reported, when possible, otherwise proxy reports were provided by parents/legal guardians. Follow‑up assessments were conducted using face‑to‑face, telephone, online, or postal methods according to family preference. Follow‑up modality was not randomized, and comparative retention or data completeness by modality was not formally evaluated.

### Measures

HRQoL was assessed with the acute PedsQL™ 4.0 suite including the parent-reported Infant Scales for ages 0–24 months and the Generic Core Scales for ages 2–18 years [11]. Fatigue was measured using the PedsQL™ Multidimensional Fatigue Scale Acute Version [12]. All versions use 3 or 5-point Likert responses (0–4), which are reverse scored and linearly transformed to a 0–100 scale, with higher scores indicating better HRQoL which is comparable across instruments. Domain and total scores are calculated as the mean of completed items. A detailed outline of their psychometric properties is reported in the methods paper [8]. For trajectory analyses, ‘return to baseline’ was defined as return to or above the individual child’s reported pre‑admission baseline score. This definition did not incorporate a minimally clinically important difference threshold. For children with severe developmental disability who were unable to self‑report, HRQoL was assessed using parent‑proxy versions of the PedsQL™ in accordance with standard instrument guidance, as detailed in the study protocol [8].

### Sample size

We powered the study to detect small-to-moderate correlations (r > 0.18) between baseline and 12‑month HRQoL (80% power, two‑sided α = 0.05). Using Fisher’s z transformation, this requires approximately 240 children with 12‑month data, allowing 20% attrition yields a recruitment target of 300 [13].

### Statistical analysis

Descriptive statistics summarize cohort characteristics and outcomes. Continuous variables are reported as mean (SD) or median (IQR) according to distribution; categorical variables as counts (percentages). All tests were 2-sided with α = 0.05. No imputation techniques were planned or used in the analysis.

For the entire analyzable sample, associations between baseline/PICU variables and HRQoL outcomes were analyzed using a machine learning approach for classification. Random Forest classifier was used to predict below-baseline HRQoL at 1-, 3-, 6- and 12-months. For each follow‑up timepoint, we created a timepoint‑specific dataset and randomly split children into training (80%) and test (20%) sets using stratification by the binary outcome (below‑baseline vs not) and a fixed random seed. Each child contributed at most one record per timepoint. To interpret the model, we utilized SHapley Additive exPlanations (SHAP). SHAP values were computed for each feature to interpret the model and understand its decision-making process. A SHAP summary plot was generated, ranking features based on their average SHAP values. The machine learning model and its interpretation were implemented using our Python package for machine learning modelling and interpretation called Helix V1.0.0.

Children with > 3 data points (Baseline, PICU discharge, and at least one other) were included in the analysis for trajectory of outcome. Due to non-normal distribution of HRQoL, Spearman correlations and Mann–Whitney tests were used to assess associations and differences between two time points. HRQoL trajectories were visualized using Sankey plots (Python v3.10, Plotly), categorizing children as “Baseline or Greater,” “Below Baseline,” or “Non-respondents” at each timepoint.

## Results

### Participant characteristics

**Fig. 1:**
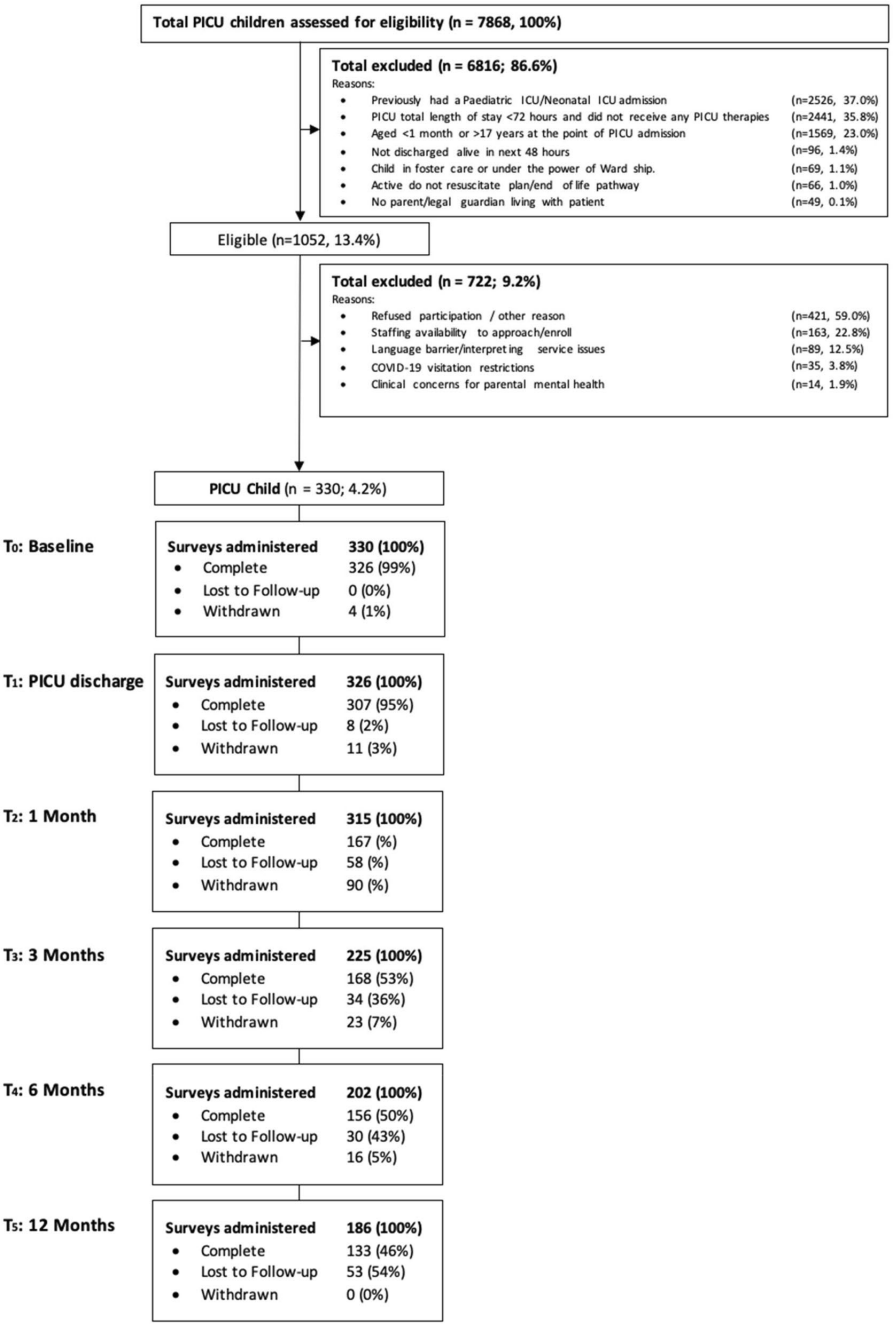
Flow diagram of children screened, enrolled/consented, and followed to 12-months post PICU discharge

The consort diagram of children screened, enrolled/consented, and followed to 12-months post-PICU discharge is provided in Fig. 1. Overall, 326 children were enrolled, with 220 contributing ≥ 3 HRQoL assessments over the 12 month data collection period. Characteristics of the samples are outlined in Table 1, with sample characteristics at each timepoint in Supplement material eTables 1 and 2. Most responses were parent-reported, though child self-reporting increased from 5.2% at PICU discharge to 24.1% at 12-months. Median age at enrolment was 2 years (interquartile range (IQR) 0 to 9), 53% were male, and 62% were White British. Most admissions were unplanned (67%). The most frequently recorded presenting diagnosis category was cardiovascular, followed by respiratory. Median PICU length of stay was 6 days (IQR 4 to 10). Mean Paediatric Index of Mortality 3 score was 0.09 (SD 0.17), corresponding to an estimated 9% risk of death. Ventilatory support was provided for a median of 5 days (IQR 3 to 9) and inotropic support for 2 days (IQR 0 to 4). Co-morbidities were recorded in 14.7% of children (median 2 conditions; IQR 1 to 3). Household deprivation scores indicated low to moderate household deprivation. Baseline Functional Status Scale data are reported descriptively. Serial Functional Status Scale assessments were not analyzed in relation to longitudinal HRQoL outcomes in this study.

**Table 1:** Total sample (n = 326) and ≥ 3‑visit subset (n = 220) characteristics at baseline

	**Total sample**	** ≥ 3‑visit subset (baseline + PICU Discharge + 1 data point)**
n	326	220
**Age, median (IQR)**	2 (0–9)	2 (0–10)
**Sex, n (%) **		
Male	174 (53.4)	111 (50.4)
**Ethnicity, n (%)**		
White British White Other Asian Pakistani Black African Asian Indian Other^a^ Mixed White/Black Caribbean Not stated/Unknown	205 (62.9) 13 (4.0) 13 (4.0) 9 (2.8) 8 (2.5) 25 (7.7) 5 (1.5) 48 (14.7)	147 (66.8) 6 (2.7) 10 (4.6) 6 (2.7) 6 (2.7) 16 (7.3) 0 (0) 29 (13.2)
**Co-morbidities, n (%)** **Median (IQR)**	48 (14.7) 2 (1–3)	30 (13.6) 1.5 (1–3)
**Household Deprivation**^**b**^**, median (IQR):** Missing (n)	1.77 (1.67–1.86) 9	1.77 (1.67–1.85) 5
**Presenting diagnosis, n (%) **Cardiovascular Respiratory Neurological Endocrine/metabolic Oncology Infection Gastrointestinal Other^c^ Unknown	69 (26.9) 51 (19.8) 32 (12.5) 27 (10.5) 19 (7.4) 17 (6.6) 15 (5.8) 27 (8.3) 69 (21.2)	46 (20.9) 36 (16.4) 22 (10.0) 21 (9.5) 11 (5.0) 11 (5.0) 12 (5.5) 18 (8.2) 43 (19.5)
**Type of admission, n (%) **		
Unplanned – otherPlanned-following surgery Unplanned-following surgery Planned-other	227 (69.6) 70 (21.5) 23 (7.1) 6 (1.8)	153 (69.5) 45 (20.4) 18 (8.2) 4 (1.8)
**PIM-3 score**^**d**^**, mean (SD)**	0.09 (0.17)	0.09 (0.17)
**Baseline Functional Status Scale,** **Median (IQR)**	326 6 (6–8)	220 6 (6–8)
**PICU Length-of-stay (days), median (IQR)**	6 (4–10)	6 (4–11)
**Days requiring ventilatory support, median (IQR)**	5 (3–9)	5 (3–9)
**Days requiring Inotropic support, median (IQR)**	2 (0–4)	2 (0–5)

**a** ^1^Ethnicity ‘Other’ includes the following ethnicities where n < 5: Black Caribbean; Mixed Other; Asian Bangladeshi; Black other; Mixed White and Asian; Chinese; White Irish. **b** Household Deprivation: UK census data 2021. The dimensions of deprivation used to classify households are indicators based on four selected household characteristics: Education (A household is classified as deprived in the education dimension if no one has at least level 2 education and no one aged 16 to 18 years is a full-time student); Employment (A household is classified as deprived in the employment dimension if any member, not a full-time student, is either unemployed or economically inactive due to long-term sickness or disability); Health (A household is classified as deprived in the health dimension if any person in the household has general health that is bad or very bad or is identified as disabled. People who have assessed their day-to-day activities as limited by long-term physical or mental health conditions or illnesses are considered disabled. This definition of a disabled person meets the harmonized standard for measuring disability and is in line with the Equality Act (2010); Housing (A household is classified as deprived in the housing dimension if the household’s accommodation is either overcrowded, in a shared dwelling, or has no central heating). Median −0.35; IQR 1 st quartile 2.46; 3rd quartile −2.35. **c** Presenting diagnosis ‘Other’ includes the following where n < 10: Bloody/lymphatic; Trauma; Urological; Musculoskeletal; Accidents and poisoning; Body wall and cavities; or Congenital** d** PIM-3 score of risk-adjusted mortality

Overall, 141/326 children (42.7%) withdrew during follow-up, most commonly at 1-month after discharge (n = 90, 64% of withdrawals; eFigure 1). From 3‑months onward, patterns of retention were similar when comparing the total enrolled sample (n = 326) and the ≥ 3‑visit analytic subset (n = 220), although absolute respondent numbers continued to decline over time. Comparison between children who provided complete data versus those who did not, showed no significant differences (eTable 3). However, later outcome estimates reflect progressively smaller samples and therefore should be interpreted with caution. All available data were included in the analyses.

### HRQoL outcomes and trajectories

**Fig. 2:**
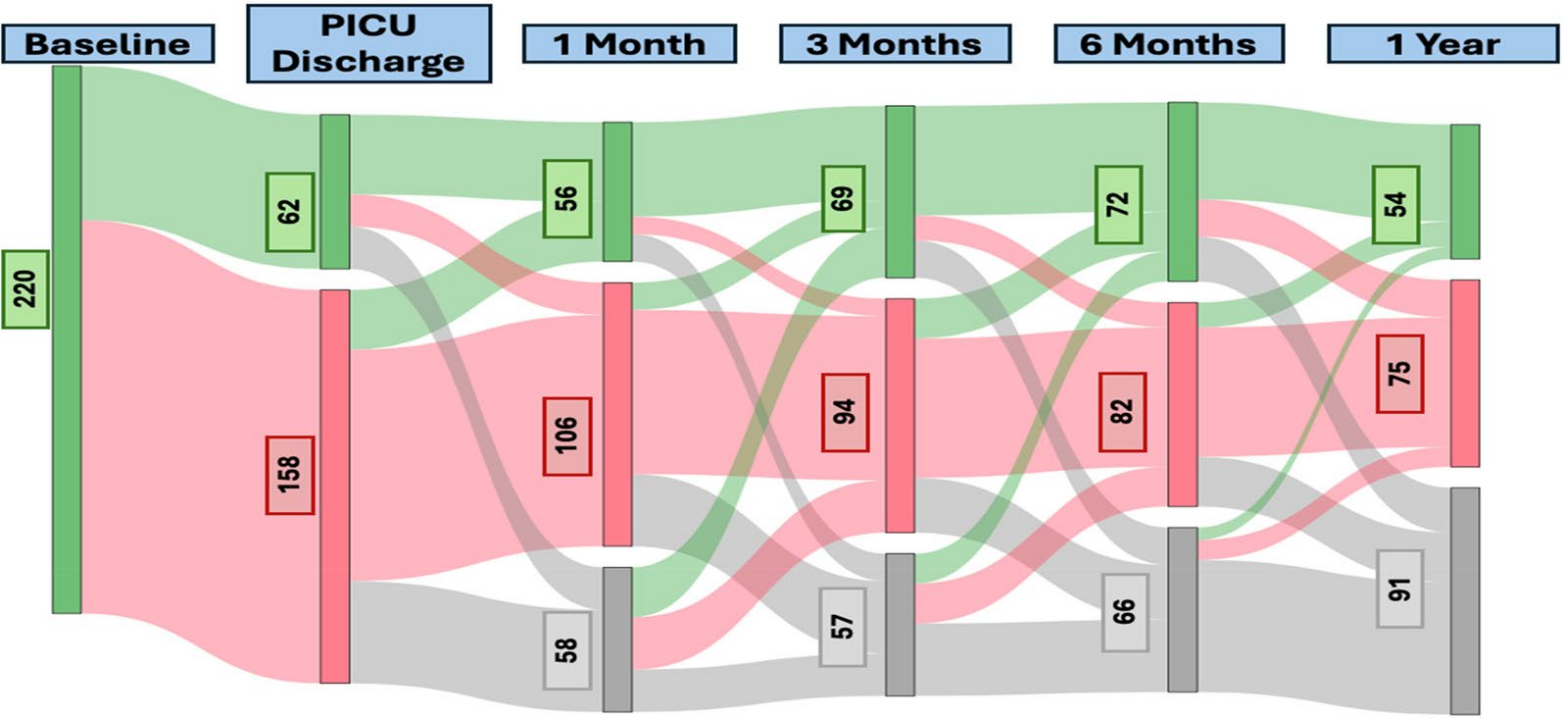
Sankey Plot showing the trajectory of quality of life categorized as equal or greater than baseline (green), below baseline (red), or non-respondents (grey) over 12-months following PICU Discharge. Numbers at each timepoint correspond with numbers of children. At PICU discharge, most children reported HRQoL below baseline (158/220, 71.8%), and 62/220 (28.2%) were at or above baseline. By 6-months, the proportion at or above baseline increased to 72/220 (32.7%) and the proportion below baseline decreased to 82/220 (37.3%), with movement in both directions between categories across follow-ups. By 12-months, non-response increased to 91/220 (41.4%). Among respondents at 12-months (n = 129), 54 (41.9%) were at or above baseline and 75 (58.1%) remained below baseline

For the primary outcome (PedsQL™ Total Health), data were available for 220 children at PICU discharge, 162 at 1-month, 163 at 3-months, 154 at 6-months, and 129 at 12-months (Table 2). Improvement in PedsQL™ Total Health score was greatest between PICU-discharge and 1-month (p < 0.001), with no clear evidence of change across later adjacent intervals (eTable 4). At PICU discharge, 158/220 (71.8%) children were below baseline HRQoL which refers to scores numerically lower than the individual child’s reported pre‑admission baseline score. Among respondents at 12‑months, 75/129 (58.1%) remained below baseline (Fig. 2).

**Table 2:** PedsQL™ Infant and core scores and Multi-dimensional Fatigue scores at baseline, PICU discharge, 1-, 3-, 6- and 12-months post-PICU discharge for ≥ 3‑visit subset

Outcome measure	Sub-scale	Baseline	PICU Discharge	1 month	3 months	6 months	12 months
**Combined PedsQL™ Infant and Core Scales,** **Mean (SD)**	Total Score	73.37 (20.99)	54.29 (23.52)	62.90 (21.73)	67.11 (20.38)	70.40 (21.34)	69.77 (22.46)
	n	220	220	162	163	154	129
	Physical Functioning Score	70.61 (30.98)	39.07 (33.93)	56.99 (31.87)	63.07 (30.86)	68.13 (29.72)	65.54 (30.06)
	n	194	194	143	143	131	100
	Physical Symptoms Score	73.22 (19.26)	73.27 (15.49)	75.93 (15.72)	76.06 (16.69)	78.78 (14.32)	79.09 (16.98)
	n	107	107	79	77	65	42
	Emotional Functioning Score	70.53 (24.73)	53.39 (25.61)	61.91 (21.69)	67.40 (19.20)	69.75 (21.97)	67.40 (22.52)
	n	219	220	162	162	153	127
	Social Functioning Score	80.75 (22.23)	68.25 (28.72)	76.69 (22.73)	77.27 (23.30)	78.13 (24.24)	77.30 (24.00)
	n	215	208	156	159	152	124
	School Functioning Score	70.36 (26.70)	52.47 (35.78)	55.57 (31.24)	57.57 (29.44)	63.42 (29.80)	65.88 (24.58)
	n	90	58	41	48	56	55
	Cognitive Functioning Score	76.03 (27.65)	64.12 (31.85)	76.24 (25.65)	75.84 (25.75)	76.83 (27.00)	72.47 (28.50)
	n	104	104	78	76	65	42
	Physical Health Score	72.64 (27.47)	45.65 (33.32)	57.28 (30.92)	63.03 (29.43)	68.49 (28.10)	68.02 (29.57)
	n	219	219	160	162	153	126
	Psychosocial Health Score	73.79 (20.94)	59.12 (23.63)	66.26 (19.45)	69.24 (18.72)	71.34 (20.70)	70.62 (20.96)
	n	219	220	162	162	154	128
**PedsQL™ Multidimensional Fatigue Scale,** **Mean (SD)**	Total Score	69.47 (26.69)	42.76 (25.17)	56.69 (23.67)	66.59 (20.81)	69.70 (23.16)	69.82 (23.57)
	n	112	108	78	81	83	85
	General Fatigue	67.54 (31.40)	34.01 (30.48)	51.19 (27.37)	63.63 (24.33)	68.93 (26.19)	69.66 (25.01)
	n	112	108	78	81	83	85
	Sleep/Rest Fatigue	64.81 (28.82)	38.86 (25.70)	57.59 (25.31)	67.70 (21.64)	71.13 (23.44)	69.51 (24.40)
	n	112	108	78	81	83	85
	Cognitive Fatigue	75.52 (26.47)	55.09 (30.88)	61.74 (27.28)	68.23 (25.75)	69.18 (29.52)	70.05 (28.74)
	n	112	108	77	80	82	85

PedsQL™ Infant Scale for 1–12 months is parent-reported and includes 36 items covering physical, symptoms, emotional, social, and cognitive domains. Normative data from USA mean total scores were: healthy 82.5 (SD 9.9), acutely ill 79.5 (SD 10.7), and chronically ill 68.0 (SD 13.9). For 13–24 months, the Infant Scale expands to 45 age-appropriate items and is scored identically. Normative data from USA mean total scores for healthy- 85.6 (SD 8.7), acutely ill- 82.2 (SD 9.2), and chronically ill- 69.9 (SD 10.4) infants. The PedsQL Generic Core Scales (v4.0) assess physical, emotional, social, and school functioning using 23 items with child self-report available from 5 years, alongside parent-proxy report. Normative data from healthy USA samples, mean total scores were 83.0 (SD 14.8) for child report and 87.6 (SD 12.3) for parent report. Fatigue was measured using the PedsQL™ Multidimensional Fatigue Scale Acute Version composed of 18 items that cover general, sleep/rest, and cognitive fatigue available in child and parent reports. Normative data from UK healthy children mean total fatigue score 81.8 (SD 12.5) with post-PICU mean score 79.6 (SD 16.3)

### Domain level HRQoL

Domain-level HRQoL showed differential recovery (Table 2). Physical Functioning and Physical Health demonstrated the largest decrement at discharge and incomplete recovery by 12-months. Physical Symptoms were unchanged at discharge and increased at 12-months (Table 2). Cognitive and emotional domains showed earlier recovery (Table 2). Cognitive Functioning declined at discharge, returned to baseline at 1-month. Emotional Functioning declined at discharge with improvement evident from discharge to 1-month (p = 0.001) and from 1 to 3-months (p = 0.017, eTable 4). Social Functioning decreased at discharge but improved by 1 month (Table 2). School Functioning declined at discharge and remained below baseline at 12-months, with no evidence of improvement between discharge and 1-month (p = 0.756).

Fatigue worsened substantially at discharge and largely normalized by 12-months (Table 2). Total Fatigue declined at discharge and recovered at 12-months. General Fatigue showed the largest decrement with recovery at 12-months. Improvements were greatest between discharge and 1-month (all p values < 0.001) and continued between 1- and 3-months, with little change thereafter.

### Baseline and PICU factors associated with impaired HRQoL

Results from the Random Forest classifier analysis to explore features associated with below-baseline HRQoL at 1-, 3-, 6- and 12-months post-discharge for the whole sample are shown in eTables 6, 7 and 8.

**Fig. 3:**
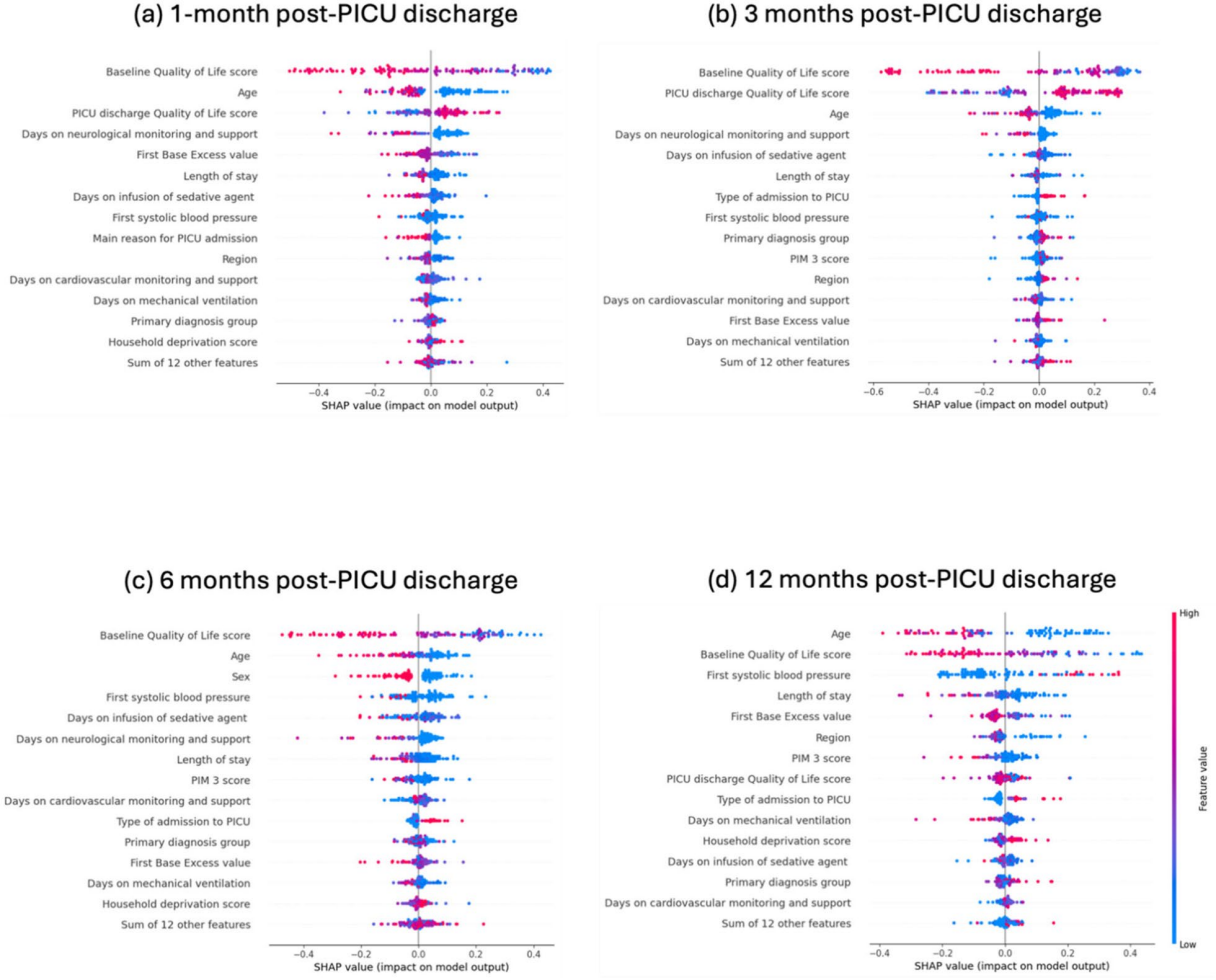
Beeswarm plot of SHapley Additive exPlanations (SHAP) values showing most important baseline and PICU variables associated with below-baseline health-related quality of life at (**a**) 1-, (**b**) 3-, (**c**) 6-, and (**d**) 12-months post-PICU. The plot illustrates the impact of each variable on the model's prediction, with each point representing an individual child's SHAP value. Higher SHAP values indicating a stronger association with below-baseline health-related quality of life. The color gradient represents the feature value (red = high value; blue = low value). Exceptions stand for the following categorical features: Sex – Red = Male, Blue = Female; Type of admission to PICU, Red = planned; Blue = unplanned. The ‘sum of 12 other features’ reflects a post‑hoc aggregation used solely for visualization. The underlying SHAP computation remains unchanged: SHAP values are calculated independently for all input features in the standard way

The SHAP beeswarm plots (Fig. 3) show at 1-month, high baseline HRQoL was the strongest feature associated with below-baseline outcomes, with SHAP values ranging from –0.4 to + 0.4. Age was the next most influential feature, with older age associated with increased risk of below-baseline outcomes (SHAP up to + 0.3). PICU discharge HRQoL was also impactful (± 0.2), where lower scores predicted worse outcomes. Additional contributors included days on neurological monitoring, first base excess, length-of-stay, and days on sedative infusion (all up to ± 0.1). First systolic blood pressure, admission reason, and geographical region had smaller effects. At 3-months, the child’s baseline and discharge HRQoL remained the dominant features (SHAP –0.6 to + 0.3 and –0.4 to + 0.2, respectively). Age continued to be influential (up to + 0.2), while days on neurological monitoring, sedative infusion, and length-of-stay each showed moderate contributions (± 0.1). Admission type, initial systolic blood pressure, and diagnosis group had lesser influence. At 6-months, the child’s baseline HRQoL remained the top feature (–0.4 to + 0.3), followed by age (+ 0.2) and sex (± 0.1). First systolic blood pressure, sedative infusion days, and neurological monitoring days each contributed up to ± 0.1. Length-of-stay, PIM-3 score, and admission type also showed moderate influence. By 12-months post-PICU discharge, age became the most important feature (–0.4 to + 0.4), surpassing baseline HRQoL (± 0.3). First systolic blood pressure, length-of-stay, and base excess (each up to ± 0.2) remained influential.

## Discussion

In this prospective, national study of children that had survived critical illness, we found a marked reduction in HRQoL at PICU discharge, which improved mainly in the first month, and then plateaued to 12-months. Physical and school domains remained impaired, while cognitive functioning recovered quickly. Attrition increased over time and limited inference at 12-months.

In the early post-discharge period, baseline HRQoL and HRQoL at PICU discharge contributed most strongly to the Random Forest model’s classification of below‑baseline HRQoL. Similar patterns have been reported in cohorts of children with bronchiolitis and sepsis, where post-discharge HRQoL reflected premorbid status and morbidity at discharge more than acute physiology alone [14, 15]. Baseline HRQoL may reflect background vulnerability and contextual factors that are not fully represented by physiological measures and that are utilized by the model when classifying outcomes. Discharge HRQoL appears to summarize the functional state at the end of the PICU admission, which the model uses when classifying early post‑discharge HRQoL outcomes. In septic shock cohorts, low baseline HRQoL predicted greater improvement, while near-normal baseline scores predicted deterioration or limited gains [16].

Systematic review evidence also suggests that worse premorbid health and neurocognitive difficulties are associated with poorer later HRQoL in PICU survivors, even after accounting for severity indices [17]. Discharge HRQoL may be most informative early and less informative later as recovery and new exposures accrue. Collectively, this framing is consistent with the PICS-p framework, in which premorbid status and contextual vulnerability interact with acute illness and PICU exposures to shape later function and HRQoL [6].

By 12‑months post‑PICU discharge, age showed the largest SHAP contribution to the model’s classification of below‑baseline HRQoL, with additional contributions from physiological and illness severity markers. The increasing SHAP contribution of age may reflect the greater salience of developmental and participation demands over time, which are more strongly expressed in HRQoL instruments as children age. This aligns with trajectory-based evidence showing that children in the most impaired recovery group were older and more likely to have pre-existing illness, suggesting that age may mark increased vulnerability to prolonged morbidity after childhood critical illness [18]. This pattern aligns with systematic review findings that, among children aged ≥ 12-months, older age, longer PICU length of stay, and higher disease-severity scores are repeatedly associated with lower HRQoL [17]. The emergence of physiological and severity markers as features of HRQoL at later follow-up is consistent with longer-term effects of early systemic stress and prolonged PICU exposure, including deconditioning and persistent symptom burden [19]. PEPaNIC study follow-up suggests that age shapes HRQoL profiles, where younger children show more growth/development problems, while older children show more role and school limitations [20]. Observational studies similarly identify longer PICU stay, multi-organ dysfunction, and invasive ventilation as risk factors for later functional decline [21, 22].

SHAP values describe how variables are used within fitted models, rather than identifying independent or causal effects. Several PICU variables included in the models, such as length of stay, duration of ventilatory support, neurological monitoring, and sedative exposure, are clinically related and likely to be correlated. When predictors are correlated, SHAP attributions may be distributed unevenly across related variables. As a result, shifts in the relative importance of features across follow up timepoints should not be interpreted as evidence of changing causal influence, but rather as differences in how correlated clinical information is represented within time specific models. Accordingly, SHAP analyzes describe model based contributions to the classification of below baseline HRQoL and do not represent causal effects or independent clinical risk estimates. Model discrimination was moderate, with test set F1 scores ranging from 0.592 to 0.743 across timepoints. Overall, these findings characterize patterns of association and highlight candidate variables for further investigation, rather than supporting individual level prognostication. Domain-level results show that post-PICU morbidity is not uniform. Physical functioning and physical health demonstrated the largest decrement at discharge and incomplete recovery by 12-months, indicating persistent below‑baseline physical functioning scores for a clinically important subgroup. Cognitive functioning returned to baseline by 1‑month and remained close to baseline at 12‑months, indicating earlier and more sustained normalization of cognitive domain scores compared with other HRQoL domains. Emotional functioning improved early and continued to improve into the 3-month timepoint, while social functioning improved more gradually. School functioning showed slower recovery, with persistent deficit at 12-months and no early improvement between discharge and 1-month, indicating an ongoing impact on participation that may not be captured by global HRQoL alone. In a large retrospective cohort, PedsQL™ scores after paediatric critical illness showed that physical functioning and school functioning contributed most to declines in overall HRQoL at 4-6 weeks follow up, whereas emotional and social domains were less impaired on average [23]. Similarly, in a self-report study 3 and 12-months after PICU discharge, children’s total PedsQL™ scores were significantly below healthy norms at 3-months but comparable by 12-months [24]. However, physical functioning scores, while improved, remained significantly lower than population norms at one year, indicating incomplete physical recovery for a substantial subgroup.

Trajectory analyses showed that the largest gains in HRQoL and fatigue occurred early after discharge, with limited additional change across later adjacent intervals. This pattern is consistent with prospective post-PICU follow-up data from Singapore [18], where health-related quality of life and functional status improved up to 3-months and then stabilized between 3 and 6-months. However, our Sankey trajectories also demonstrated heterogeneity, including movement in both directions between categories across follow-ups. These movements illustrate heterogeneity in observed HRQoL trajectories but do not imply directional progression or regression at the individual level. Trajectory modelling in the Singapore cohort similarly identified distinct recovery phenotypes (mild, moderate, severe), with a small subgroup showing persistent or worsening impairment by 6-months [18]. These findings support needs-based follow-up, with early post-discharge assessment and referral. In the OCEANIC cohort, functional status declined initially but returned to near baseline by 1-month and remained stable, suggesting that basic functional abilities recover more rapidly than broader quality of life measures. This aligns with prior evidence indicating that functional recovery trajectories vary by condition but generally trend toward improvement or remain static [25]. Cognitive recovery was relatively rapid and sustained, potentially reflecting developmental resilience and informal rehabilitative processes. In contrast, emotional and social functioning improved more gradually, consistent with literature showing that psychosocial recovery often lags behind physical recovery [26]. School functioning may remain persistently affected, highlighting the need for long term educational support. Fatigue followed a similar trajectory to HRQoL, with early impairments, particularly in general and sleep related fatigue, improving by 6 to 12-months. These findings contrast with Colville et al., [27], who reported no significant fatigue differences compared to the general population, possibly due to differences in length of stay or pandemic related factors. This highlights the importance of addressing sleep hygiene and energy management in post PICU care [28].

Random Forest model performance varied by timepoint in this cohort, reflecting differences in available sample size and outcome distribution across follow‑up assessments. SHAP analyses showed baseline and discharge HRQoL dominated early prediction, while age became most influential by 12 months. This shift suggests determinants change as recovery progresses and supports combining baseline context with exposure markers in follow-up planning. These models still require external validation before informing individual-level decisions. For clinicians, SHAP‑based findings should be interpreted as exploratory and hypothesis‑generating. SHAP identifies which variables contributed most to model classification within this dataset but does not estimate effect sizes, quantify risk, or support individual‑level prognostication. These results should therefore be considered alongside clinical judgement and complementary analytic approaches when informing post‑PICU follow‑up strategies.

Non-response increased at 12 months, consistent with HRQoL studies where attrition is common and often under-characterized [29]. Later estimates may rely on smaller and potentially healthier samples. This could underestimate morbidity, so we interpret 12-month findings cautiously and note that families with worse health or greater social disadvantage may therefore be under-represented.

### Limitations

This study has several limitations. First, attrition increased over time, reducing the 12‑month sample and raising the possibility of non-response bias. Second, baseline HRQoL was reported retrospectively (2 weeks pre-admission), which may introduce recall bias. Third, most outcomes relied on parent-proxy report, with increasing child self-report over time. As such, mode and respondent changes may contribute to measurement variability.

## Conclusion

This multicenter prospective study describes health‑related quality of life trajectories during the first year after PICU discharge in a general paediatric intensive care population. HRQoL declined markedly at discharge, improved largely within the first post‑discharge month, and showed little further recovery thereafter. At one year, many children remained below their pre‑PICU baseline, with persistent impairment most evident in physical and school functioning.

Explainable machine‑learning analyses identified baseline and discharge HRQoL, age, and markers of PICU exposure as variables contributing to model classification of below‑baseline HRQoL across follow‑up timepoints. These findings are hypothesis‑generating and reflect model‑based associations rather than causal effects or individual‑level risk estimates. Collectively, the results inform future research and post‑PICU follow‑up planning and should be interpreted alongside clinical judgement and complementary analytic approaches.

## Supplementary Information

Additional file1

## Data Availability

The datasets generated and/or analyzed during the current study are not publicly available due ongoing analysis by the OCEANIC study team but are available from the corresponding author on reasonable request.
