# Supplementary material for: One year quality of life outcomes in critically ill children: a multicenter prospective cohort study
Source: Crit Care. 2026 May 16;30:361. doi: 10.1186/s13054-026-06084-9 (PMC13348625; doi:10.1186/s13054-026-06084-9)
Supplement: Supplementary file 1 — Additional file1 [file 13054_2026_6084_MOESM1_ESM.docx]

**ELECTRONIC SUPPLEMENTARY MATERIAL**

**eFigure 1: Participant retention and forward attrition across study visits at screening/enrolment, baseline, PICU discharge, 1-, 3-, 6-, 12- months post-PICU of whole sample (n=326) and subset cohort (n=220)**

**
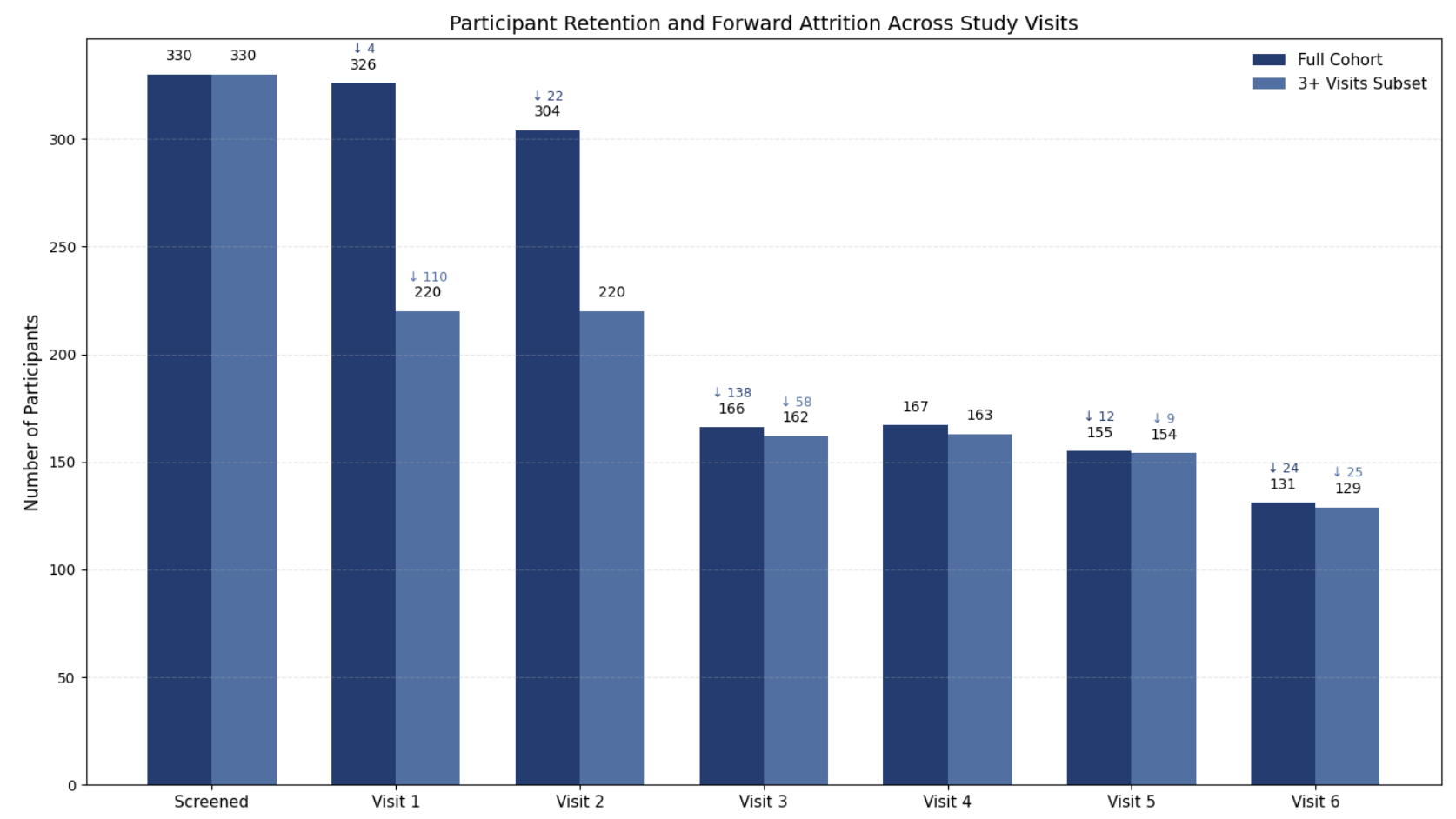
**

**eTable 1: Whole sample (n=326) detailed characteristics at baseline, PICU discharge, 1-, 3-, 6- and 12- months post-PICU discharge.**

|  | **Baseline** | **PICU Discharge** | **1 month** | **3 months** | **6 months** | **12 months** |  |
| --- | --- | --- | --- | --- | --- | --- | --- |
| Child | n=326 | n=307 | n=167 | n=168 | n=156 | n=133 |  |
| **Age, median (IQR)** | 2 (0-9) | 2 (0-9) | 2 (0-9) | 2 (0-11) | 2 (1-10) | 3 (1-11) |  |
| **Sex**, **n (%)** Male | 174 (53.4) | 164 (53.4) | 86 (51.5) | 94 (56.0) | 83 (53.2) | 75 (56.4) |  |
| **Reporting, n (%)** Parent-reported  Self-reported | 309 (94.8)  17 (5.2) | 273 (88.9) 34 (11.1) | 133 (79.6) 34 (20.4) | 133 (79.2) 35 (20.8) | 124 (79.5) 32 (20.5) | 101 (75.9) 32 (24.1) |  |
| **Ethnicity, n (%)** White British  White Other  Asian Pakistani  Black African  Asian Indian  Other  Mixed White/Black Caribbean  Asian other  Black Caribbean  Mixed Oher  Asian Bangladeshi  Black other  Mixed White and Asian  Chinese  White Irish  Not stated/Unknown | 205 (62.88)  13 (3.99)  13 (3.99)  9 (2.76)  8 (2.45)  8 (2.45)  5 (1.53)  3 (0.92)  3 (0.92)  3 (0.92)  3 (0.92)  2 (0.61)  1 (0.31)  1 (0.31)  1 (0.31)  48 (14.72) | 193 (62.87) 13 (4.23) 13 (4.23) 9 (2.93)  7 (2.28) 8 (2.61) 4 (1.3) 3 (0.98)  2 (0.65) 3 (0.98) 3 (0.98) 2 (0.65) 1 (0.33) 1 (0.33) 1 (0.33)  44 (14.33) | 116 (69.46)  4 (2.4) 7 (4.19)  1 (0.6)  3 (1.8) 4 (2.4) 0 (0.0)  1 (0.6) 2 (1.2) 1 (0.6)  0 (0.0) 1 (0.6) 1 (0.6) 1 (0.6) 1 (0.6)  24 (14.37) | 112 (66.67) 4 (2.38) 6 (3.57) 4 (2.38) 4 (2.38) 4 (2.38)  0 (0.0) 2 (1.19) 2 (1.19) 2 (1.19) 1 (0.6) 1 (0.6) 1 (0.6) 1 (0.6)  0 (0.0)  24 (14.29) | 111 (71.15) 3 (1.92) 5 (3.21) 2 (1.28)  2 (1.28)  5 (3.21) 0 (0.0) 2 (1.28) 2 (1.28) 1 (0.64) 1 (0.64) 1 (0.64)  0 (0.0) 1 (0.64) 1 (0.64)  19 (12.08) | 94 (70.68)  3 (2.26) 4 (3.01) 3 (2.26) 3 (2.26) 3 (2.26)  0 (0.0) 2 (1.5) 2 (1.5) 1 (0.75) 1 (0.75)  0 (0.0) 1 (0.75)  0 (0.0)  0 (0.0)  16 (12.03) |  |
| **Presenting diagnosis, n (%)** Cardiovascular  Respiratory  Neurological  Endocrine / metabolic  Oncology  Infection  Gastrointestinal  Bloody / lymphatic  Trauma  Urological  Musculoskeletal  Accidents and poisoning  Body wall and cavities  Congenital  Unknown | 69 (26.9) 51 (19.8) 32 (12.5) 27 (10.5) 19 (7.4) 17 (6.6) 15 (5.8) 8 (3.1) 5 (2.0) 4 (1.6) 4 (1.6) 3 (1.2) 2 (0.8) 1 (0.4)  69 (21.2) | 69 (22.48) 46 (14.98) 30 (9.77) 26 (8.47) 19 (6.19) 15 (4.89) 15 (4.89) 8 (2.61) 6 (1.95)  3 (0.98) 4 (1.30) 2 (0.65) 2 (0.65) 1 (0.33)  61 (19.87) | 35 (26.32) 25 (18.8) 19 (14.29) 14 (10.53) 7 (5.26)  10 (7.52) 7 (5.26) 7 (5.26) 3 (2.26)  1 (0.75)  1 (0.75) 2 (1.5) 2 (1.5) 0 (0.0)  34 (20.36) | 39 (27.86) 24 (17.14) 15 (10.71)  18 (12.86) 11 (7.86) 7 (5.0)  8 (5.71) 7 (5.0) 4 (2.86) 1 (0.71) 1 (0.71)  2 (1.43) 2 (1.43) 1 (0.71)  44 (26.19) | 31 (24.6) 26 (20.63) 16 (12.7) 15 (11.9) 9 (7.14) 9 (7.14) 6 (4.76) 5 (3.97) 2 (1.59)  1 (0.79) 2 (1.59)  1 (0.79) 2 (1.59) 1 (0.79)  30 (19.23) | 25 (22.73) 22 (20.0) 12 (10.91)  16 (14.55)  8 (7.27) 9 (8.18) 5 (4.55) 5 (4.55) 2 (1.82)  1 (0.91) 2 (1.82) 1 (0.91) 1 (0.91) 1 (0.91) 23 (17.29) |  |
| **Type of admission, n (%)** Unplanned – other  Planned - following surgery  Unplanned - following surgery  Planned - other | 227 (69.6) 70 (21.5) 23 (7.1) 6 (1.8) | 211 (68.7) 69 (22.5) 21 (6.8) 6 (2.0) | 116 (69.5) 33 (19.8) 16 (9.6) 2 (1.2) | 112 (66.7) 40 (23.8) 13 (7.7) 3 (1.8) | 110 (70.5) 32 (20.5) 11 (7.1) 3 (1.9) | 96 (72.2) 25 (18.8) 10 (7.5) 2 (1.5) |  |
| **PICU Length-of-stay (days), median (IQR)** | 6 (4-10) | 6 (4-10) | 7 (4-11) | 7 (4-12) | 6 (4-11) | 6 (4-12) |  |
| **PIM-3 score^a^, mean (SD)** | 0.09 (0.17) | 0.09 (0.17) | 0.08 (0.15) | 0.10 (0.17) | 0.08 (0.15) | 0.09 (0.17) |  |
| **Days requiring ventilatory support, median (IQR)** | 5 (3-9) | 5 (3-9) | 6 (3-9) | 5 (3-10) | 5 (3-9) | 5 (3-10) |  |
| **Days requiring Inotropic support, median (IQR)** | 2 (0-4) | 2 (0-4) | 2 (0-5) | 2 (0-5) | 2 (0-5) | 2 (0-5) |  |
| **Co-morbidities, n (%)**  **Median (IQR)** | 48 (14.7)  2 (1-3) | 44 (14.3)  1.5 (1-3) | 25 (15.0)  2 (1-3) | 22 (13.1)  2 (1-3) | 25 (16.0)  1 (1-3) | 20 (15.0)  1 (1-3) |  |
| **Household Deprivation^b^, median (IQR):**  Missing (n)  Count of quintile:  0.0-0.2  0.2-0.4  0.4-0.6  0.6-0.8  0.8-1.0 | 1.77 (1.67-1.86)  9  1.63 (1.6-1.65)  1.7 (1.68-1.72)  1.78 (1.76-1.8)  1.85 (1.82-1.86)  1.91 (1.91-1.93) | 1.77 (1.68-1.86)  8  1.63 (1.6-1.65)  1.7 (1.68-1.72)  1.78 (1.76-1.8)  1.85 (1.82-1.86)  1.91 (1.91-1.93) | 1.77 (1.67-1.86)  3  1.63 (1.61-1.65)  1.69 (1.67-1.71)  1.77 (1.76-1.8)  1.85 (1.82-1.86)  1.91 (1.91-1.92) | 1.77 (1.67-1.84)  4  1.63 (1.59-1.66)  1.7 (1.67-1.72)  1.78 (1.76-1.79)  1.82 (1.81-1.84)  1.91 (1.9-1.91) | 1.76 (1.67-1.85)  4  1.63 (1.58-1.65)  1.69 (1.67-1.71)  1.77 (1.76-1.78)  1.84 (1.81-1.86)  1.91 (1.9-1.93) | 1.77 (1.67-1.84)  2  1.63 (1.6-1.66)  1.69 (1.67-1.71)  1.77 (1.76-1.78)  1.81 (1.81-1.84)  1.91 (1.89-1.92) |  |
| **a** PIM-3 score of risk-adjusted mortality. **b** Household Deprivation: UK census data 2021. The dimensions of deprivation used to classify households are indicators based on four selected household characteristics: Education (A household is classified as deprived in the education dimension if no one has at least level 2 education and no one aged 16 to 18 years is a full-time student); Employment (A household is classified as deprived in the employment dimension if any member, not a full-time student, is either unemployed or economically inactive due to long-term sickness or disability); Health (A household is classified as deprived in the health dimension if any person in the household has general health that is bad or very bad or is identified as disabled. People who have assessed their day-to-day activities as limited by long-term physical or mental health conditions or illnesses are considered disabled. This definition of a disabled person meets the harmonised standard for measuring disability and is in line with the Equality Act (2010); Housing (A household is classified as deprived in the housing dimension if the household's accommodation is either overcrowded, in a shared dwelling, or has no central heating). Median -0.35; IQR 1^st^ quartile 2.46; 3^rd^ quartile -2.35. | | | | | | | |

**eTable 2: Subset cohort (n=220) detailed characteristics at baseline, PICU discharge, 1-, 3-, 6- and 12- months post-PICU discharge.**

|  | **Baseline** | **PICU Discharge** | **1 Month** | **3 Months** | **6 Months** | **12 Months** |
| --- | --- | --- | --- | --- | --- | --- |
| Child | n=220 | n=220 | n=162 | n=163 | n=154 | n=129 |
| **Age, median (IQR)** | 2 (0-10) | 2 (0-10) | 1.5 (0-9) | 2 (0-10) | 1.5 (0-10) | 2 (0-10) |
| **Sex**, **n (%)** Male | 111 (50.45) | 111 (50.45) | 82.0 (50.62) | 90.0 (55.21) | 81.0 (52.6) | 71.0 (55.04) |
| **Ethnicity, n (%)** White British  White Other  Asian Pakistani  Black African  Asian Indian  Other  Asian other  Black Caribbean  Mixed Oher  Asian Bangladeshi  Black other  Mixed White and Asian  Chinese  White Irish  Not stated/Unknown | 147.0 (66.82)  6.0 (2.73)  10.0 (4.55)  6.0 (2.73)  6.0 (2.73)  5.0 (2.27)  2.0 (0.91)  2.0 (0.91)  2.0 (0.91)  1.0 (0.45)  1.0 (0.45)  1.0 (0.45)  1.0 (0.45)  1.0 (0.45)  29.0 (13.18) | 147.0 (66.82)  6.0 (2.73)  10.0 (4.55)  6.0 (2.73)  6.0 (2.73)  5.0 (2.27)  2.0 (0.91)  2.0 (0.91)  2.0 (0.91)  1.0 (0.45)  1.0 (0.45)  1.0 (0.45)  1.0 (0.45)  1.0 (0.45)  29.0 (13.18) | 112.0 (69.14)  4.0 (2.47)  7.0 (4.32)  1.0 (0.62)  3.0 (1.85)  4.0 (2.47)  1.0 (0.62)  2.0 (1.23)  1.0 (0.62)  0 (0.00)  1.0 (0.62)  1.0 (0.62)  1.0 (0.62)  1.0 (0.62)  23.0 (14.2) | 108.0 (66.26)  4.0 (2.45)  6.0 (3.68)  4.0 (2.45)  4.0 (2.45)  4.0 (2.45)  2.0 (1.23)  2.0 (1.23)  2.0 (1.23)  1.0 (0.61)  1.0 (0.61)  1.0 (0.61)  1.0 (0.61)  0 (0.00)  23.0 (14.11) | 110.0 (71.43)  3.0 (1.95)  5.0 (3.25)  2.0 (1.3)  2.0 (1.3)  5.0 (3.25)  2.0 (1.3)  2.0 (1.3)  1.0 (0.65)  1.0 (0.65)  0 (0.00)  1.0 (0.65)  1.0 (0.65)  1.0 (0.65)  18.0 (11.69) | 92.0 (71.32)  2.0 (1.55)  4.0 (3.1)  3.0 (2.33)  3.0 (2.33)  3.0 (2.33)  2.0 (1.55)  2.0 (1.55)  1.0 (0.78)  1.0 (0.78)  0 (0.00)  1.0 (0.78)  0 (0.00)  0 (0.00)  15.0 (11.63) |
| **Presenting diagnosis, n (%)** Cardiovascular  Respiratory  Neurological  Endocrine / metabolic  Oncology  Infection  Gastrointestinal  Bloody / lymphatic  Trauma  Urological  Musculoskeletal  Accidents and poisoning  Body wall and cavities  Congenital  Unknown | 46.0 (20.91)  36.0 (16.36)  22.0 (10.0)  21.0 (9.55)  11.0 (5.0)  11.0 (5.0)  12.0 (5.45)  7.0 (3.18)  4.0 (1.82)  1.0 (0.45)  2.0 (0.91)  1.0 (0.45)  2.0 (0.91)  1.0 (0.45)  43 (19.55) | 46.0 (20.91)  36.0 (16.36)  22.0 (10.0)  21.0 (9.55)  11.0 (5.0)  11.0 (5.0)  12.0 (5.45)  7.0 (3.18)  4.0 (1.82)  1.0 (0.45)  2.0 (0.91)  1.0 (0.45)  2.0 (0.91)  1.0 (0.45)  43 (19.55) | 34.0 (20.99)  25.0 (15.43)  19.0 (11.73)  13.0 (8.02)  7.0 (4.32)  10.0 (6.17)  7.0 (4.32)  7.0 (4.32)  3.0 (1.85)  1.0 (0.62)  1.0 (0.62)  1.0 (0.62)  2.0 (1.23)  0 (0.00)  32 (19.75) | 38.0 (23.31)  23.0 (14.11)  15.0 (9.2)  17.0 (10.43)  11.0 (6.75)  7.0 (4.29)  8.0 (4.91)  7.0 (4.29)  4.0 (2.45)  1.0 (0.61)  1.0 (0.61)  1.0 (0.61)  2.0 (1.23)  1.0 (0.61)  27 (16.56) | 30.0 (19.48)  26.0 (16.88)  16.0 (10.39)  15.0 (9.74)  9.0 (5.84)  9.0 (5.84)  6.0 (3.9)  5.0 (3.25)  2.0 (1.3)  1.0 (0.65)  2.0 (1.3)  1.0 (0.65)  2.0 (1.3)  1.0 (0.65)  29 (18.83) | 24.0 (18.6)  22.0 (17.05)  12.0 (9.3)  15.0 (11.63)  8.0 (6.2)  9.0 (6.98)  5.0 (3.88)  5.0 (3.88)  2.0 (1.55)  1.0 (0.78)  2.0 (1.55)  1.0 (0.78)  1.0 (0.78)  1.0 (0.78)  21 (16.28) |
| **Type of admission, n (%)** Unplanned – other  Planned - following surgery  Unplanned - following surgery  Planned - other | 153.0 (69.55)  45.0 (20.45)  18.0 (8.18)  4.0 (1.82) | 153.0 (69.55)  45.0 (20.45)  18.0 (8.18)  4.0 (1.82) | 113.0 (69.75)  32.0 (19.75)  15.0 (9.26)  2.0 (1.23) | 109.0 (66.87)  38.0 (23.31)  13.0 (7.98)  3.0 (1.84) | 109.0 (70.78)  31.0 (20.13)  11.0 (7.14)  3.0 (1.95) | 93.0 (72.09)  24.0 (18.6)  10.0 (7.75)  2.0 (1.55) |
| **PICU Length-of-stay (days), median (IQR)** | 6 (4-11) | 6 (4-11) | 7 (4-11) | 7 (4-12) | 6 (4-11.75) | 6 (4-12) |
| **PIM-3 score^a^, mean (SD)** | 0.095 (0.17) | 0.095 (0.17) | 0.08 (0.15) | 0.09 (0.17) | 0.08 (0.15) | 0.09 (0.17) |
| **Days requiring ventilatory support, median (IQR)** | 5 (3-9) | 5 (3-9) | 6 (3-9) | 5 (3-10) | 5 (3-9) | 5 (3-10) |
| **Days requiring Inotropic support, median (IQR)** | 2 (0-5) | 2 (0-5) | 2 (0-5) | 2 (0-5) | 2 (0-5) | 2 (0-5) |
| **Co-morbidities, n (%)**  **Median (IQR)** | 30 (13.64)  1.5 (1-3) | 30 (13.64)  1.5 (1-3) | 24 (14.81)  1.5 (1-3) | 21 (12.88)  2 (1-3) | 25 (16.23)  1 (1-3) | 20 (15.5)  1 (1-3) |
| **Household Deprivation^b^, median (IQR):**  Missing (n)  Count of quintile:  0.0-0.2  0.2-0.4  0.4-0.6  0.6-0.8  0.8-1.0 | 1.77 (1.67-1.85)  5  1.62 (1.59-1.65)  1.69 (1.67-1.72)  1.78 (1.76-1.79)  1.84 (1.81-1.86)  1.91 (1.91-1.93) | 1.77 (1.67-1.85)  5  1.62 (1.59-1.65)  1.69 (1.67-1.72)  1.78 (1.76-1.79)  1.84 (1.81-1.86)  1.91 (1.91-1.93) | 1.76 (1.67-1.86)  3  1.63 (1.61-1.65)  1.69 (1.67-1.71)  1.77 (1.76-1.79)  1.85 (1.82-1.87)  1.91 (1.91-1.92) | 1.77 (1.67-1.84)  3  1.63 (1.59-1.66)  1.69 (1.67-1.71)  1.77 (1.76-1.79)  1.82 (1.81-1.84)  1.91 (1.9-1.91) | 1.76 (1.67-1.85)  4  1.62 (1.58-1.65)  1.69 (1.67-1.71)  1.76 (1.74-1.77)  1.82 (1.8-1.85)  1.91 (1.9-1.92) | 1.77 (1.67-1.84)  2  1.63 (1.6-1.65)  1.69 (1.67-1.71)  1.76 (1.76-1.77)  1.81 (1.8-1.84)  1.91 (1.89-1.92) |
| a PIM-3 score of risk-adjusted mortality. b Household Deprivation: UK census data 2021. The dimensions of deprivation used to classify households are indicators based on four selected household characteristics: Education (A household is classified as deprived in the education dimension if no one has at least level 2 education and no one aged 16 to 18 years is a full-time student); Employment (A household is classified as deprived in the employment dimension if any member, not a full-time student, is either unemployed or economically inactive due to long-term sickness or disability); Health (A household is classified as deprived in the health dimension if any person in the household has general health that is bad or very bad or is identified as disabled. People who have assessed their day-to-day activities as limited by long-term physical or mental health conditions or illnesses are considered disabled. This definition of a disabled person meets the harmonised standard for measuring disability and is in line with the Equality Act (2010); Housing (A household is classified as deprived in the housing dimension if the household's accommodation is either overcrowded, in a shared dwelling, or has no central heating). Median -0.35; IQR 1st quartile 2.46; 3rd quartile -2.35. | | | | | | |

**eTable 3: Difference in samples (Kruskal Wallis Test) in child baseline and PICU characteristics who provided usable data versus those who did not, at baseline, PICU discharge, 1-, 3-, 6-, 12- months post-PICU.**

| Variable / Category | Baseline | PICU Discharge | 1 month | 3 months | 6 months | 12 months |
| --- | --- | --- | --- | --- | --- | --- |
| Age | 0.431  (n=220 vs 106) | 0.410  (n=220 vs 84) | 0.118 (n=162 vs 4) | 0.671 (n=163 vs 4) | NA  (n=154 vs 1) | 0.077 (n=129 vs 2) |
| Sex | 0.155 (n=220 vs 106) | 0.124  (n=220 vs 84) | 0.621  (n=162 vs 4) | 0.630 (n=163 vs 4) | 1.000 (n=154 vs 1) | 0.503 (n=129 vs 2) |
| Male | 0.155  (111 vs 63) | 0.124  (111 vs 51) | 0.621  (82 vs 3) | 0.630  (90 vs 3) | 1.000  (81 vs 1) | 0.503  (71 vs 2) |
| Female | 0.155  (109 vs 43) | 0.124  (109 vs 33) | 0.621  (80 vs 1) | 0.630  (73 vs 1) | 1.000  (73 vs 0) | 0.503  (58 vs 0) |
| Reporting | 0.596  (n=220 vs 106) | 0.554  (n=220 vs 84) | 0.186  (n=162 vs 4) | 0.194 (n=163 vs 4) | 1.000 (n=154 vs 1) | 1.000 (n=129 vs 2) |
| Parent-reported | 0.596  (207 vs 102) | 0.554  (193 vs 76) | 0.186 (130 vs 2) | 0.194 (130 vs 2) | 1.000 (122 vs 1) | 1.000 (96 vs 2) |
| Self-reported | 0.596  (13 vs 4) | 0.554  (27 vs 8) | 0.186 (32 vs 2) | 0.194 (33 vs 2) | 1.000 (32 vs 0) | 1.000 (33 vs 0) |
| Ethnicity | 0.168  (n=220 vs 106) | 0.068  (n=220 vs 84) | 1.000  (n=162 vs 4) | 1.000 (n=163 vs 4) | 0.880 (n=154 vs 1) | 0.992 (n=129 vs 2) |
| Asian Bangladeshi | 0.248  (1 vs 2) | 0.186  (1 vs 2) | 1.000  (0 vs 0) | 1.000  (1 vs 0) | 1.000  (1 vs 0) | 1.000  (1 vs 0) |
| Asian Indian | 1.000  (6 vs 2) | 0.678  (6 vs 1) | 1.000  (3 vs 0) | 1.000  (4 vs 0) | 1.000  (2 vs 0) | 1.000  (3 vs 0) |
| Asian Pakistani | 0.559  (10 vs 3) | 1.000  (10 vs 3) | 1.000  (7 vs 0) | 1.000  (6 vs 0) | 1.000  (5 vs 0) | 1.000  (4 vs 0) |
| Asian other | 1.000  (2 vs 1) | 1.000  (2 vs 1) | 1.000  (1 vs 0) | 1.000  (2 vs 0) | 1.000  (2 vs 0) | 1.000  (2 vs 0) |
| Black African | 1.000  (6 vs 3) | 0.711  (6 vs 3) | 1.000  (1 vs 0) | 1.000  (4 vs 0) | 1.000  (2 vs 0) | 1.000  (3 vs 0) |
| Black Caribbean | 1.000  (2 vs 1) | 1.000  (2 vs 0) | 1.000  (2 vs 0) | 1.000  (2 vs 0) | 1.000  (2 vs 0) | 1.000  (2 vs 0) |
| Black other | 0.545  (1 vs 1) | 0.477  (1 vs 1) | 1.000  (1 vs 0) | 1.000  (1 vs 0) | 1.000  (0 vs 0) | 1.000  (0 vs 0) |
| Chinese | 1.000  (1 vs 0) | 1.000  (1 vs 0) | 1.000  (1 vs 0) | 1.000  (1 vs 0) | 1.000  (1 vs 0) | 1.000  (0 vs 0) |
| Mixed White and Asian | 1.000  (1 vs 0) | 1.000  (1 vs 0) | 1.000  (1 vs 0) | 1.000  (1 vs 0) | 1.000  (1 vs 0) | 1.000  (1 vs 0) |
| Mixed White and Black Caribbean | 0.003  (0 vs 5) | 0.006  (0 vs 4) | 1.000  (0 vs 0) | 1.000  (0 vs 0) | 1.000  (0 vs 0) | 1.000  (0 vs 0) |
| Mixed other | 1.000  (2 vs 1) | 1.000  (2 vs 1) | 1.000  (1 vs 0) | 1.000  (2 vs 0) | 1.000  (1 vs 0) | 1.000  (1 vs 0) |
| Other | 0.718  (5 vs 3) | (1 vs 3) | 1.000  (1 vs 0) | 1.000  (4 vs 0) | 1.000  (5 vs 0) | 1.000  (3 vs 0) |
| Unknown | 0.157  (29 vs 19) | 0.121  (29 vs 15) | 0.421  (23 vs 1) | 0.419  (23 vs 1) | 0.110  (18 vs 1) | 0.203  (15 vs 1) |
| White British | 0.038  (147 vs 58) | 0.024  (147 vs 44) | 1.000  (112 vs 3) | 1.000  (108 vs 3) | 0.290  (110 vs 0) | 0.498  (92 vs 1) |
| White Irish | 1.000  (1 vs 0) | 1.000  (1 vs 0) | 1.000  (1 vs 0) | 1.000  (0 vs 0) | 1.000  (1 vs 0) | 1.000  (0 vs 0) |
| White other | 0.129  (6 vs 7) | 0.099  (6 vs 6) | 1.000  (4 vs 0) | 1.000  (4 vs 0) | 1.000  (3 vs 0) | 1.000  (2 vs 0) |
| Presenting diagnosis | 0.506  (n=177 vs 80) | 0.590  (n=177 vs 68) | <0.001 (n=130 vs 2) | 0.022 (n=136 vs 3) | NA  (n=125 vs 0) | 0.951 (n=108 vs 1) |
| Accidents and poisoning | 0.229  (1 vs 2) | 0.479  (1 vs 1) | 0.030  (1 vs 1) | 0.043  (1 vs 1) | 1.000  (1 vs 0) | 1.000  (1 vs 0) |
| Blood / lymphatic | 0.441  (7 vs 1) | 0.450  (7 vs 1) | 1.000  (7 vs 0) | 1.000  (7 vs 0) | 1.000  (5 vs 0) | 1.000  (5 vs 0) |
| Body wall and cavities | 1.000  (2 vs 0) | 1.000  (2 vs 0) | 1.000  (2 vs 0) | 1.000  (2 vs 0) | 1.000  (2 vs 0) | 1.000  (1 vs 0) |
| Cardiovascular | 0.651  (46 vs 23) | 0.267  (46 vs 23) | 1.000  (34 vs 0) | 0.562  (38 vs 0) | 1.000  (30 vs 0) | 1.000  (24 vs 0) |
| Congenital | 1.000  (1 vs 0) | 1.000  (1 vs 0) | 1.000  (0 vs 0) | 1.000  (1 vs 0) | 1.000  (1 vs 0) | 1.000  (1 vs 0) |
| Endocrine / metabolic | 0.381  (21 vs 6) | 0.362  (21 vs 5) | 0.202  (13 vs 1) | 0.342  (17 vs 1) | 1.000  (15 vs 0) | 0.147  (15 vs 1) |
| Gastrointestinal | 0.404  (12 vs 3) | 0.766  (12 vs 3) | 1.000  (7 vs 0) | 1.000  (8 vs 0) | 1.000  (6 vs 0) | 1.000  (5 vs 0) |
| Infection | 0.787  (11 vs 6) | 1.000  (11 vs 4) | 1.000  (10 vs 0) | 1.000  (7 vs 0) | 1.000  (9 vs 0) | 1.000  (9 vs 0) |
| Musculoskeletal | 0.591  (2 vs 2) | 0.308  (2 vs 2) | 1.000  (1 vs 0) | 1.000  (1 vs 0) | 1.000  (2 vs 0) | 1.000  (2 vs 0) |
| Neurological | 1.000  (22 vs 10) | 1.000  (22 vs 8) | 1.000  (19 vs 0) | 1.000  (15 vs 0) | 1.000  (16 vs 0) | 1.000  (12 vs 0) |
| Oncology | 0.308  (11 vs 8) | 0.181  (11 vs 8) | 1.000  (7 vs 0) | 1.000  (11 vs 0) | 1.000  (9 vs 0) | 1.000  (8 vs 0) |
| Respiratory | 0.866  (36 vs 15) | 0.364  (36 vs 10) | 1.000  (25 vs 0) | 0.436  (23 vs 1) | 1.000  (26 vs 0) | 1.000  (22 vs 0) |
| Trauma | 1.000  (4 vs 1) | 1.000  (4 vs 1) | 1.000  (3 vs 0) | 1.000  (4 vs 0) | 1.000  (2 vs 0) | 1.000  (2 vs 0) |
| Urological | 0.091  (1 vs 3) | 0.187  (1 vs 2) | 1.000  (1 vs 0) | 1.000  (1 vs 0) | 1.000  (1 vs 0) | 1.000  (1 vs 0) |
| Type of admission | 0.672  (n=220 vs 106) | 0.341 (n=220 vs 84) | 0.602 (n=162 vs 4) | 0.933 (n=163 vs 4) | 0.938 (n=154 vs 1) | 0.857 (n=129 vs 2) |
| Planned - following surgery | 0.565  (45 vs 25) | 0.219  (45 vs 23) | 1.000  (32 vs 0) | 1.000  (38 vs 1) | 1.000  (31 vs 0) | 1.000  (24 vs 0) |
| Planned - other | 1.000  (4 vs 2) | 0.670  (4 vs 2) | 1.000  (2 vs 0) | 1.000  (3 vs 0) | 1.000  (3 vs 0) | 1.000  (2 vs 0) |
| Unplanned - following surgery | 0.356  (18 vs 5) | 0.208  (18 vs 3) | 0.336  (15 vs 1) | 1.000  (13 vs 0) | 1.000  (11 vs 0) | 1.000  (10 vs 0) |
| Unplanned - other | 1.000  (153 vs 74) | 0.679  (153 vs 56) | 1.000  (113 vs 3) | 1.000  (109 vs 3) | 1.000  (109 vs 1) | 1.000  (93 vs 2) |
| PICU Length-of-stay | 0.702  (n=220 vs 106) | 0.597 (n=220 vs 84) | 0.428  (n=162 vs 4) | 0.318 (n=163 vs 4) | NA  (n=154 vs 1) | 0.341 (n=129 vs 2) |
| PIM-3 score | 0.612  (n=220 vs 106) | 0.840 (n=220 vs 84) | 0.790  (n=162 vs 4) | 0.714 (n=163 vs 4) | NA  (n=154 vs 1) | 0.680 (n=129 vs 2) |
| Days requiring ventilatory support | 0.504  (n=220 vs 106) | 0.652 (n=220 vs 84) | 0.646 (n=162 vs 4) | 0.858 (n=163 vs 4) | NA  (n=154 vs 1) | 0.611 (n=129 vs 2) |
| Days requiring Inotropic support | 0.081  (n=220 vs 106) | 0.263 (n=220 vs 84) | 0.692 (n=162 vs 4) | 0.205 (n=163 vs 4) | NA  (n=154 vs 1) | 0.350 (n=129 vs 2) |
| Co-morbidities | 0.866  (n=30 vs 18) | 0.839  (n=30 vs 14) | NA  (n=24 vs 1) | NA  (n=21 vs 1) | NA  (n=25 vs 0) | NA  (n=20 vs 0) |
| Baseline Household Deprivation (IMD quintile) | 0.241  (n=215 vs 102) | 0.158 (n=215 vs 81) | 0.050  (n=159 vs 4) | 0.025 (n=160 vs 3) | <0.001 (n=150 vs 1) | 0.001 (n=127 vs 2) |
| 0-20% | 1.000 | 1.000 | 1.000 | 1.000 | 1.000 | 1.000 |
| 20-40% | 1.000 | 1.000 | 1.000 | 1.000 | 1.000 | 1.000 |
| 40-60% | 1.000 | 1.000 | 1.000 | 1.000 | 1.000 | 1.000 |
| 60-80% | 1.000 | 1.000 | 1.000 | 1.000 | 1.000 | 1.000 |
| 80-100% | 1.000 | 1.000 | 1.000 | 1.000 | 1.000 | 1.000 |

**eTable 4: The difference (Kruskal Wallis test) and correlation (Spearman’s Rho) in the trajectory of recovery between consecutive timepoints for PedsQL™ Infant and core scales for ≥3‑visit subset.** Statistically significant (p 0.05) results presented.

| **Test** | **Outcome measure** | **Sub-scale** | **PICU Discharge - 1 month post PICU discharge** | **1 month post PICU discharge - 3 months post PICU discharge** | **3 months post PICU discharge - 6 months post PICU discharge** | **6 months post PICU discharge - 12 months post PICU discharge** |
| --- | --- | --- | --- | --- | --- | --- |
| **Kruskal Wallis Test, p-value** | **Combined PedsQL™ Infant and Core Scales** | Total Health | <0.001 |  |  |  |
|  |  | n | 220–162 | 162–163 | 163–154 | 154–129 |
|  |  | Physical Functioning | <0.001 |  |  |  |
|  |  | n | 194–143 | 143–143 | 143–131 | 131–100 |
|  |  | Physical Symptoms |  |  |  |  |
|  |  | n | 107–79 | 79–77 | 77–65 | 65–42 |
|  |  | Emotional Functioning | 0.001 | 0.017 |  |  |
|  |  | n | 220–162 | 162–162 | 162–153 | 153–127 |
|  |  | Social Functioning | 0.011 |  |  |  |
|  |  | n | 208–156 | 156–159 | 159–152 | 152–124 |
|  |  | School Functioning |  |  |  |  |
|  |  | n | 58–41 | 41–48 | 48–56 | 56–55 |
|  |  | Cognitive Functioning | 0.024 |  |  |  |
|  |  | n | 104–78 | 78–76 | 76–65 | 65–42 |
|  |  | Physical Health | 0.001 |  |  |  |
|  |  | n | 219–160 | 160–162 | 162–153 | 153–126 |
|  |  | Psychosocial Health | 0.004 |  |  |  |
|  |  | n | 220–162 | 162–162 | 162–154 | 154–128 |
|  | **Multidimensional Fatigue Scale** | Total score | <0.001 | 0.007 |  |  |
|  |  | n | 108–78 | 78–81 | 81–83 | 83–85 |
|  |  | General Fatigue | <0.001 | 0.003 |  |  |
|  |  | n | 108–78 | 78–81 | 81–83 | 83–85 |
|  |  | Sleep/rest fatigue | <0.001 | 0.014 |  |  |
|  |  | n | 108–78 | 78–81 | 81–83 | 83–85 |
|  |  | Cognitive Fatigue |  |  |  |  |
|  |  | n | 108–77 | 77–80 | 80–82 | 82–85 |
| **Spearman’s Rho,**  **Correlation coefficient (p-value)** | **Combined PedsQL™ Infant and Core Scales** | Total Health | 0.66 (<0.001) | 0.79 (<0.001) | 0.81 (<0.001) | 0.80 (<0.001) |
|  |  | n | 220–162 | 162–163 | 163–154 | 154–129 |
|  |  | Physical Functioning | 0.58 (<0.001) | 0.76 (<0.001) | 0.73 (<0.001) | 0.70 (<0.001) |
|  |  | n | 194–143 | 143–143 | 143–131 | 131–100 |
|  |  | Physical Symptoms | 0.30 (0.007) | 0.73 (<0.001) | 0.72 (<0.001) | 0.79 (<0.001) |
|  |  | n | 107–79 | 79–77 | 77–65 | 65–42 |
|  |  | Emotional Functioning | 0.58 (<0.001) | 0.62 (<0.001) | 0.64 (<0.001) | 0.62 (<0.001) |
|  |  | n | 220–162 | 162–162 | 162–153 | 153–127 |
|  |  | Social Functioning | 0.38 (<0.001) | 0.50 (<0.001) | 0.73 (<0.001) | 0.78 (<0.001) |
|  |  | n | 208–156 | 156–159 | 159–152 | 152–124 |
|  |  | School Functioning |  | 0.63 (<0.001) | 0.54 (<0.001) | 0.51 (<0.001) |
|  |  | n | 58–41 | 41–48 | 48–56 | 56–55 |
|  |  | Cognitive Functioning | 0.52 (<0.001) | 0.54 (<0.001) | 0.63 (<0.001) | 0.81 (<0.001) |
|  |  | n | 104–78 | 78–76 | 76–65 | 65–42 |
|  |  | Physical Health | 0.62 (<0.001) | 0.78 (<0.001) | 0.72 (<0.001) | 0.74 (<0.001) |
|  |  | n | 219–160 | 160–162 | 162–153 | 153–126 |
|  |  | Psychosocial Health | 0.55 (<0.001) | 0.71 (<0.001) | 0.77 (<0.001) | 0.75 (<0.001) |
|  |  | n | 220–162 | 162–162 | 162–154 | 154–128 |
|  | **Multidimensional Fatigue Scale** | Total score | 0.55 (<0.001) | 0.8 (<0.001) | 0.8 (<0.001) | 0.88 (<0.001) |
|  |  | n | 108 - 78 | 78 - 81 | 81 - 83 | 83 - 85 |
|  |  | General Fatigue | 0.53 (<0.001) | 0.74 (<0.001) | 0.8 (<0.001) | 0.82 (<0.001) |
|  |  | n | 108 - 78 | 78 - 81 | 81 - 83 | 83 - 85 |
|  |  | Sleep/rest fatigue | 0.38 (<0.001) | 0.66 (<0.001) | 0.66 (<0.001) | 0.83 (<0.001) |
|  |  | n | 108 - 78 | 78 - 81 | 81 - 83 | 83 - 85 |
|  |  | Cognitive Fatigue | 0.64 (<0.001) | 0.78 (<0.001) | 0.68 (<0.001) | 0.76 (<0.001) |
|  |  | n | 108 - 77 | 77 - 80 | 80 - 82 | 82 - 85 |

**eTable 5: Random Forest classification results**

Hyperparameter tuning using grid search was conducted to optimise the model, focusing on parameters such as bootstrap sampling, class weights, criterion, maximum depth, minimum samples per leaf, and the number of estimators. The model's performance against the training data was evaluated using K-fold cross-validation (K=5), focusing on accuracy and F1 scores. Confusion matrices were generated to calculate precision, recall, sensitivity, and specificity.

| **Data** | **Set** | | **accuracy** | **F1 score** | **Precision** | | | **Recall** | **AUC** |
| --- | --- | --- | --- | --- | --- | --- | --- | --- | --- |
| **1 month** | Test | | 0.734 ± 0.086 | 0.592 ± 0.125 | 0.627 ± 0.114 | | | 0.578 ± 0.163 | 0.696 ± 0.097 |
|  | Train | | 0.911 ± 0.011 | 0.878 ± 0.015 | 0.825 ± 0.022 | | | 0.939 ± 0.027 | 0.918 ± 0.013 |
| **3 months** | Test | | 0.797 ± 0.044 | 0.743 ± 0.080 | 0.767 ± 0.053 | | 0.745 ± 0.176 | | 0.789 ± 0.062 |
|  | Train | | 0.929 ± 0.009 | 0.915 ± 0.011 | 0.903 ± 0.019 | | 0.927 ± 0.022 | | 0.928 ± 0.010 |
| **6 months** | Test | | 0.677 ± 0.076 | 0.642 ± 0.108 | 0.656 ± 0.081 | | | 0.638 ± 0.145 | 0.676 ± 0.079 |
|  | Train | | 0.911 ± 0.021 | 0.908 ± 0.022 | 0.879 ± 0.022 | | | 0.940 ± 0.028 | 0.913 ± 0.021 |
| **12 months** | Test | 0.722 ± 0.131 | | 0.649 ± 0.172 | 0.664 ± 0.146 | 0.653 ± 0.220 | | | 0.712 ± 0.139 |
|  | Train | 0.911 ± 0.010 | | 0.897 ± 0.012 | 0.862 ± 0.019 | 0.936 ± 0.029 | | | 0.914 ± 0.011 |

**eTable 6: Whole sample (N=326) Pre-PICU [baseline] - PedsQL™ Infant and Core Scales, -Multidimensional Fatigue Scale and -** **Functional status scale at each data collection point.**

|  | **Baseline** | **PICU Discharge** | **1 month** | **3 months** | **6 months** | **12 months** |
| --- | --- | --- | --- | --- | --- | --- |
| Child | N=326 | n=307 | n=167 | n=168 | n=156 | n=133 |
| **Pre-PICU [baseline] combined PedsQL™ Infant and Core Scales, mean (SD)**  Total Health Score  Physical Functioning Score  Physical Symptoms Score  Emotional Functioning Score  Social Functioning Score  School Functioning Score  Cognitive Functioning Score  Physical Health Score  Psychosocial Health Score | 72.11 (21.93)  68.95 (31.45)  73.12 (19.18)  69.72 (25.24)  79.72 (23.77)  68.29 (29.19)  75.72 (27.19)  70.47 (28.66)  72.96 (21.97) | 53.71 (24.02)  38.97 (34.18)  72.31 (15.78)  51.89 (26.32)  67.10 (29.97)  50.10 (35.87)  61.92 (32.58)  45.93 (33.57)  57.92 (24.68) | 63.12 (21.50)  49.65 (65.28)  75.93 (15.52)  61.88 (21.48)  76.96 (22.60)  55.55 (30.86)  76.60 (25.47)  57.63 (30.64)  66.38 (19.28) | 66.93 (20.71)  54.81 (67.89)  76.06 (16.54)  67.01 (19.63)  77.31 (23.61)  56.67 (30.01)  76.30 (25.59)  62.90 (29.46)  69.03 (19.12) | 70.42 (21.20)  56.14 (72.57)  78.67 (14.29)  69.56 (21.89)  78.25 (24.15)  63.42 (29.80)  77.24 (26.74)  68.52 (27.95)  71.34 (20.58) | 70.11 (22.21)  53.26 (73.84)  79.86 (16.66)  67.50 (22.19)  77.69 (23.82)  66.13 (24.43)  73.26 (27.75)  68.46 (29.24)  70.90 (20.71) |
| **Multidimensional Fatigue Scale, mean (SD)**  Total Score  General Fatigue  Sleep/Rest Fatigue  Cognitive Fatigue | n=166  68.57 (26.88)  66.63 (31.30)  63.96 (29.54)  74.77 (28.05) | n=150  43.94 (25.43)  35.59 (31.30)  39.01 (26.33)  56.94 (31.53) | n=82  57.18 (23.35)  51.53 (26.80)  57.98 (24.91)  62.45 (27.01) | n=84  66.14 (21.92)  63.29 (25.20)  67.11 (22.62)  67.82 (26.60) | n=82  69.72 (23.30)  68.95 (26.34)  71.23 (23.57)  69.11 (29.70) | n=84  69.15 (23.29)  68.94 (24.71)  68.80 (24.19)  69.44 (28.58) |
| **Pre-PICU [baseline] Functional status scale, median (IQR)**  Total score  Feeding  Mental status  Motor function  Communication  Respiratory  Sensory | 6 (6-8)  1 (1-1)  1 (1-2)  1 (1-1)  1 (1-1)  1 (1-1)  1 (1-1) | 9 (6-12)  2 (1-3)  1 (1-2)  1 (1-3)  1 (1-2)  1 (1-2)  1 (1-1) | 6 (6-8)  1 (1-2)  1 (1-1)  1 (1-1)  1 (1-1)  1 (1-1)  1 (1-1) | 6 (6-8)  1 (1-1)  1 (1-1)  1 (1-1)  1 (1-1)  1 (1-1)  1 (1-1) | 6 (6-8)  1 (1-1)  1 (1-1)  1 (1-1)  1 (1-1)  1 (1-1)  1 (1-1) | 6 (6-7)  1 (1-1)  1 (1-1)  1 (1-1)  1 (1-1)  1 (1-1)  1 (1-1) |
| PedsQL Infant Scale for 1–12 months is parent-reported and includes 36 items covering physical, symptoms, emotional, social, and cognitive domains. Normative data from USA mean total scores were: healthy 82.5 (SD 9.9), acutely ill 79.5 (SD 10.7), and chronically ill 68.0 (SD 13.9).  For 13–24 months, the Infant Scale expands to 45 age-appropriate items and is scored identically. Normative data from USA mean total scores for healthy- 85.6 (SD 8.7), acutely ill- 82.2 (SD 9.2), and chronically ill- 69.9 (SD 10.4) infants.  The PedsQL Generic Core Scales (v4.0) assess physical, emotional, social, and school functioning using 23 items with child self-report available from 5 years, alongside parent-proxy report. Normative data from healthy USA samples, mean total scores were 83.0 (SD 14.8) for child report and 87.6 (SD 12.3) for parent report.  Fatigue was measured using the PedsQL™ Multidimensional Fatigue Scale Acute Version composed of 18 items that cover general, sleep/rest, and cognitive fatigue available in child and parent reports. Normative data from UK healthy children mean total fatigue score 81.8 (SD 12.5) with post-PICU mean score 79.6 (SD 16.3). | | | | | | |

**eTable 7: Whole sample (N=326) PedsQL™ Infant (<1 year, 1-2 years), core (2-18 years) and combined scores, multidimensional fatigue scale, and Functional Status Scale scores at baseline, PICU discharge, 1-, 3-, 6- and 12- months post-PICU discharge**

| **Outcome measure** | **Sub-scale** | **Baseline** | **PICU Discharge** | **1 month** | **3 months** | **6 months** | **12 months** |
| --- | --- | --- | --- | --- | --- | --- | --- |
| **Combined PedsQL™ Infant and Core Scales,**  **Mean (SD)** | Total Health Score | 72.11 (21.9) | 53.71 (24.03) | 63.28 (21.42) | 66.93 (20.71) | 70.51 (21.16) | 70.11 (22.21) |
|  | n | 326 | 307 | 169 | 168 | 156 | 133 |
|  | Physical Functioning Score | 68.96 (31.5) | 38.97 (34.19) | 57.52 (31.58) | 63.46 (30.62) | 68.32 (29.51) | 66.04 (29.68) |
|  | n | 282 | 267 | 148 | 147 | 134 | 104 |
|  | Physical Symptoms Score | 73.12 (19.2) | 72.31 (15.78) | 75.95 (15.43) | 76.10 (16.54) | 78.65 (14.18) | 79.86 (16.66) |
|  | n | 158 | 152 | 82 | 79 | 68 | 45 |
|  | Emotional Functioning Score | 69.72 (25.2) | 51.90 (26.33) | 62.00 (21.37) | 67.01 (19.63) | 69.58 (21.82) | 67.50 (22.19) |
|  | n | 325 | 306 | 169 | 167 | 156 | 131 |
|  | Social Functioning Score | 79.72 (23.8) | 67.10 (29.97) | 76.10 (22.54) | 77.31 (23.61) | 78.40 (24.14) | 77.70 (23.82) |
|  | n | 321 | 290 | 162 | 164 | 155 | 128 |
|  | School Functioning Score | 68.29 (29.2) | 50.10 (35.88) | 55.81 (30.54) | 56.67 (30.02) | 63.42 (29.80) | 66.13 (24.43) |
|  | n | 136 | 85 | 43 | 50 | 56 | 56 |
|  | Cognitive Functioning Score | 75.72 (27.2) | 61.92 (32.58) | 76.89 (25.44) | 76.30 (25.59) | 77.57 (26.68) | 73.26 (27.75) |
|  | n | 153 | 147 | 81 | 78 | 68 | 45 |
|  | Psychosocial Health score | 72.96 (21.96) | 57.92 (24.67) | 66.38 (19.28) | 69.03 (19.12) | 71.34 (20.58) | 70.90 (20.71) |
|  | n | 325 | 307 | 167 | 167 | 156 | 132 |
|  | Physical Health Score | 70.48 (28.7) | 45.93 (33.57) | 57.89 (30.54) | 62.91 (29.46) | 68.63 (27.90) | 68.46 (29.24) |
|  | n | 325 | 306 | 166 | 167 | 156 | 130 |
| **PedsQL™ Infant (<1 year) Scales,**  **Mean (SD)** | Total Health Score | 73.50 (17.46) | 64.02 (19.19) | 73.13 (14.02) | 74.29 (12.82) | 78.05 (15.06) | 79.17 (29.46) |
|  | n | 114 | 111 | 59 | 53 | 38 | 3 |
|  | Physical Functioning Score | 71.40 (25.45) | 52.31 (27.81) | 73.59 (19.52) | 75.49 (20.91) | 79.39 (22.22) | 56.25 (61.87) |
|  | n | 114 | 110 | 59 | 53 | 38 | 3 |
|  | Physical Symptoms Score | 72.72 (18.02) | 73.09 (15.61) | 73.84 (15.06) | 72.74 (16.79) | 79.41 (14.52) |  |
|  | n | 114 | 111 | 58 | 53 | 38 |  |
|  | Emotional Functioning Score | 71.55 (21.55) | 58.95 (22.69) | 68.17 (15.91) | 71.08 (13.72) | 72.75 (16.45) | 90.62 (13.26) |
|  | n | 113 | 110 | 59 | 53 | 38 | 3 |
|  | Social Functioning Score | 80.69 (24.74) | 69.23 (28.23) | 84.27 (18.03) | 83.73 (21.21) | 86.68 (20.81) |  |
|  | n | 111 | 108 | 58 | 53 | 38 |  |
|  | Cognitive Functioning Score | 77.75 (26.94) | 66.37 (31.72) | 76.97 (24.89) | 76.18 (22.83) | 79.93 (25.80) |  |
|  | n | 110 | 107 | 57 | 53 | 38 |  |
|  | Psychosocial Health score | 74.51 (19.79) | 62.58 (22.88) | 72.79 (15.25) | 74.65 (13.39) | 76.97 (15.19) | 85.00 (21.21) |
|  | n | 113 | 111 | 59 | 53 | 38 | 3 |
|  | Physical Health Score | 72.31 (18.57) | 65.80 (17.60) | 73.76 (15.19) | 73.84 (16.44) | 79.40 (16.25) | 71.88 (39.77) |
|  | n | 114 | 111 | 59 | 53 | 38 | 3 |
| **PedsQL™ Infant (1-2 year) Scales,**  **Mean (SD)** | Total Health Score | 70.58 (22.20) | 53.48 (20.99) | 74.05 (17.05) | 78.07 (17.81) | 75.15 (17.87) | 77.90 (16.65) |
|  | n | 44 | 41 | 24 | 27 | 30 | 45 |
|  | Physical Functioning Score | 68.37 (29.92) | 40.97 (29.88) | 72.77 (22.94) | 79.63 (23.39) | 76.72 (21.26) | 80.09 (19.69) |
|  | n | 44 | 41 | 23 | 27 | 30 | 44 |
|  | Physical Symptoms Score | 74.15 (22.11) | 70.20 (16.25) | 81.22 (15.73) | 82.86 (13.96) | 77.69 (14.17) | 80.26 (16.64) |
|  | n | 44 | 41 | 23 | 26 | 29 | 44 |
|  | Emotional Functioning Score | 66.52 (25.41) | 53.05 (23.33) | 67.10 (19.23) | 71.47 (17.92) | 70.07 (19.18) | 72.18 (18.00) |
|  | n | 44 | 41 | 24 | 26 | 30 | 45 |
|  | Social Functioning Score | 77.50 (24.79) | 55.64 (31.19) | 82.72 (19.17) | 89.05 (18.06) | 87.07 (18.00) | 87.73 (14.61) |
|  | n | 44 | 41 | 23 | 25 | 29 | 44 |
|  | Cognitive Functioning Score | 70.54 (27.45) | 50.00 (32.25) | 75.66 (27.40) | 76.56 (31.17) | 73.71 (27.99) | 74.22 (27.31) |
|  | n | 43 | 40 | 23 | 25 | 29 | 44 |
|  | Psychosocial Health score | 70.16 (23.03) | 52.64 (23.08) | 72.72 (18.20) | 75.63 (19.13) | 74.05 (19.54) | 76.14 (17.98) |
|  | n | 44 | 41 | 24 | 26 | 30 | 45 |
|  | Physical Health Score | 71.20 (23.81) | 55.01 (20.75) | 76.73 (17.65) | 81.41 (17.64) | 77.13 (17.22) | 80.18 (16.83) |
|  | n | 44 | 41 | 23 | 27 | 30 | 44 |
| **PedsQL™ core Scales,**  **Mean (SD)** | Total Health Score | 71.57 (24.51) | 46.39 (25.28) | 52.96 (22.27) | 59.08 (22.29) | 65.41 (23.36) | 65.57 (23.68) |
|  | n | 168 | 155 | 86 | 88 | 88 | 85 |
|  | Physical Functioning Score | 66.92 (36.63) | 25.62 (36.17) | 37.28 (31.96) | 47.43 (32.09) | 57.55 (32.93) | 55.72 (31.12) |
|  | n | 124 | 116 | 65 | 67 | 65 | 58 |
|  | Emotional Functioning Score | 69.33 (27.44) | 46.59 (28.33) | 55.98 (23.92) | 63.24 (22.36) | 67.91 (24.85) | 64.17 (23.74) |
|  | n | 168 | 155 | 84 | 88 | 86 | 83 |
|  | Social Functioning Score | 79.67 (22.92) | 68.81 (30.37) | 69.94 (24.50) | 69.94 (24.10) | 71.64 (25.37) | 72.41 (26.16) |
|  | n | 166 | 141 | 79 | 86 | 87 | 83 |
|  | School Functioning Score | 68.29 (29.19) | 50.10 (35.88) | 55.56 (30.86) | 56.67 (30.02) | 63.42 (29.80) | 66.13 (24.43) |
|  | n | 136 | 85 | 42 | 50 | 56 | 56 |
|  | Psychosocial Health score | 72.65 (23.09) | 55.98 (25.90) | 60.07 (20.20) | 63.70 (20.60) | 67.90 (22.52) | 67.56 (21.59) |
|  | n | 168 | 155 | 84 | 88 | 87 | 84 |
|  | Physical Health Score | 69.04 (34.88) | 29.20 (36.35) | 40.68 (32.49) | 50.50 (32.88) | 60.81 (32.37) | 62.25 (32.42) |
|  | n | 167 | 154 | 82 | 87 | 87 | 84 |
| **Multidimensional Fatigue Scale,**  **Mean (SD)** | Total Score | 68.57 (26.89) | 43.94 (25.44) | 57.55 (23.32) | 66.14 (21.92) | 69.70 (23.16) | 69.82 (23.43) |
|  | n | 166 | 150 | 84 | 84 | 83 | 86 |
|  | General Fatigue | 66.63 (31.30) | 35.59 (31.30) | 52.04 (26.84) | 63.29 (25.20) | 68.93 (26.20) | 69.67 (24.87) |
|  | n | 166 | 150 | 84 | 84 | 83 | 86 |
|  | Sleep/Rest Fatigue | 63.96 (29.54) | 39.01 (26.34) | 58.23 (24.83) | 67.11 (22.62) | 71.13 (23.44) | 69.43 (24.27) |
|  | n | 166 | 150 | 84 | 84 | 83 | 86 |
|  | Cognitive Fatigue | 74.77 (28.06) | 56.94 (31.53) | 62.80 (26.83) | 67.82 (26.60) | 69.20 (29.52) | 70.11 (28.58) |
|  | n | 166 | 150 | 83 | 83 | 82 | 86 |
| **Functional Status Scale,**  **Median (IQR)** | Total score | 6 (6–8) | 9 (6–12) | 6 (6–8) | 6 (6–8) | 6 (6–8) | 6 (6–7) |
|  | n | 326 | 310 | 177 | 174 | 161 | 137 |
|  | Feeding | 1 (1–1) | 2 (1–3) | 1 (1–2) | 1 (1–1) | 1 (1–1) | 1 (1–1) |
|  | n | 317 | 295 | 166 | 170 | 152 | 137 |
|  | Mental status | 1 (1–2) | 1 (1–2) | 1 (1–1) | 1 (1–1) | 1 (1–1) | 1 (1–1) |
|  | n | 324 | 302 | 167 | 170 | 151 | 135 |
|  | Motor function | 1 (1–1) | 1 (1–3) | 1 (1–1) | 1 (1–1) | 1 (1–1) | 1 (1–1) |
|  | n | 314 | 295 | 166 | 168 | 152 | 136 |
|  | Communication | 1 (1–1) | 1 (1–2) | 1 (1–1) | 1 (1–1) | 1 (1–1) | 1 (1–1) |
|  | n | 318 | 300 | 167 | 170 | 151 | 136 |
|  | Respiratory | 1 (1–1) | 1 (1–2) | 1 (1–1) | 1 (1–1) | 1 (1–1) | 1 (1–1) |
|  | n | 314 | 296 | 166 | 170 | 152 | 136 |
|  | Sensory | 1 (1–1) | 1 (1–1) | 1 (1–1) | 1 (1–1) | 1 (1–1) | 1 (1–1) |
|  | n | 320 | 298 | 167 | 170 | 151 | 136 |
| PedsQL Infant Scale for 1–12 months is parent-reported and includes 36 items covering physical, symptoms, emotional, social, and cognitive domains. Normative data from USA mean total scores were: healthy 82.5 (SD 9.9), acutely ill 79.5 (SD 10.7), and chronically ill 68.0 (SD 13.9).  For 13–24 months, the Infant Scale expands to 45 age-appropriate items and is scored identically. Normative data from USA mean total scores for healthy- 85.6 (SD 8.7), acutely ill- 82.2 (SD 9.2), and chronically ill- 69.9 (SD 10.4) infants.  The PedsQL Generic Core Scales (v4.0) assess physical, emotional, social, and school functioning using 23 items with child self-report available from 5 years, alongside parent-proxy report. Normative data from healthy USA samples, mean total scores were 83.0 (SD 14.8) for child report and 87.6 (SD 12.3) for parent report.  Fatigue was measured using the PedsQL™ Multidimensional Fatigue Scale Acute Version composed of 18 items that cover general, sleep/rest, and cognitive fatigue available in child and parent reports. Normative data from UK healthy children mean total fatigue score 81.8 (SD 12.5) with post-PICU mean score 79.6 (SD 16.3). | | | | | | | |

**eTable 8: The difference (Kruskal Wallis test) and correlation (Spearman’s Rho) in the trajectory of recovery between consecutive timepoints for PedsQL™ Infant and core scales for whole sample (N=326)**

| **Test** | **Outcome measure** | **Sub-scale** | **PICU Discharge - 1 month post PICU discharge** | **1 month post PICU discharge - 3 months post PICU discharge** | **3 months post PICU discharge - 6 months post PICU discharge** | **6 months post PICU discharge - 12 months post PICU discharge** |
| --- | --- | --- | --- | --- | --- | --- |
| **Kruskal Wallis Test, p-value** | **Combined PedsQL™ Infant and Core Scales** | Total Health | <0.001 | 0.149 | 0.137 | 0.944 |
|  |  | n | 307–167 | 167–168 | 168–156 | 156–133 |
|  |  | Physical Functioning | <0.001 | 0.119 | 0.200 | 0.551 |
|  |  | n | 267–147 | 147–147 | 147–133 | 133–104 |
|  |  | Physical Symptoms | 0.120 | 0.794 | 0.519 | 0.524 |
|  |  | n | 152–81 | 81–79 | 79–67 | 67–45 |
|  |  | Emotional Functioning | <0.001 | 0.044 | 0.298 | 0.475 |
|  |  | n | 306–167 | 167–167 | 167–155 | 155–131 |
|  |  | Social Functioning | 0.005 | 0.721 | 0.620 | 0.636 |
|  |  | n | 290–160 | 160–164 | 164–154 | 154–128 |
|  |  | School Functioning | 0.535 | 0.953 | 0.242 | 0.796 |
|  |  | n | 85–42 | 42–50 | 50–56 | 56–56 |
|  |  | Cognitive Functioning | 0.002 | 0.950 | 0.710 | 0.428 |
|  |  | n | 147–80 | 80–78 | 78–67 | 67–45 |
|  |  | Physical Health | 0.001 | 0.136 | 0.088 | 0.895 |
|  |  | n | 307–167 | 167–167 | 167–156 | 156–132 |
|  |  | Psychosocial Health | 0.002 | 0.241 | 0.235 | 0.816 |
|  |  | n | 306-164 | 164-167 | 167-155 | 155-130 |
|  | **Multidimensional Fatigue Scale** | Total score | <0.001 | 0.031 | 0.373 | 0.998 |
|  |  | n | 150–82 | 82–84 | 84–82 | 82–84 |
|  |  | General Fatigue | 0.001 | 0.018 | 0.217 | 0.921 |
|  |  | n | 150–82 | 82–84 | 84–82 | 82–84 |
|  |  | Sleep/rest fatigue | <0.001 | 0.046 | 0.317 | 0.652 |
|  |  | n | 150–82 | 82–84 | 84–82 | 82–84 |
|  |  | Cognitive Fatigue | 0.301 | 0.246 | 0.510 | 0.858 |
|  |  | n | 150–81 | 81–83 | 83–81 | 81–84 |
| **Spearman’s Rho,**  **Correlation coefficient (p-value)** | **Combined PedsQL™ Infant and Core Scales** | Total Health | 0.66 (<0.001) | 0.79 (<0.001) | 0.81 (<0.001) | 0.78 (<0.001) |
|  |  | n | 307–167 | 167–168 | 168–156 | 156–133 |
|  |  | Physical Functioning | 0.58 (<0.001) | 0.76 (<0.001) | 0.72 (<0.001) | 0.69 (<0.001) |
|  |  | n | 267–147 | 147–147 | 147–133 | 133–104 |
|  |  | Physical Symptoms | 0.30 (0.007) | 0.73 (<0.001) | 0.73 (<0.001) | 0.75 (<0.001) |
|  |  | n | 152–81 | 81–79 | 79–67 | 67–45 |
|  |  | Emotional Functioning | 0.58 (<0.001) | 0.61 (<0.001) | 0.64 (<0.001) | 0.61 (<0.001) |
|  |  | n | 306–167 | 167–167 | 167–155 | 155–131 |
|  |  | Social Functioning | 0.38 (<0.001) | 0.50 (<0.001) | 0.73 (<0.001) | 0.78 (<0.001) |
|  |  | n | 290–160 | 160–164 | 164–154 | 154–128 |
|  |  | School Functioning | 0.34 (0.064) | 0.64 (<0.001) | 0.54 (0.001) | 0.51 (0.001) |
|  |  | n | 85–42 | 42–50 | 50–56 | 56–56 |
|  |  | Cognitive Functioning | 0.52 (<0.001) | 0.55 (<0.001) | 0.64 (<0.001) | 0.81 (<0.001) |
|  |  | n | 147–80 | 80–78 | 78–67 | 67–45 |
|  |  | Physical Health | 0.62 (<0.001) | 0.78 (<0.001) | 0.72 (<0.001) | 0.74 (<0.001) |
|  |  | n | 307–167 | 167–167 | 167–156 | 156–132 |
|  |  | Psychosocial Health | 0.54 (<0.001) | 0.70 (<0.001) | 0.77 (<0.001) | 0.74 (<0.001) |
|  |  | n | 306–164 | 164–167 | 167–155 | 155–130 |
|  | **Multidimensional Fatigue Scale** | Total score | 0.57 (<0.001) | 0.80 (<0.001) | 0.80 (<0.001) | 0.88 (<0.001) |
|  |  | n | 150–82 | 82–84 | 84–82 | 82–84 |
|  |  | General Fatigue | 0.53 (<0.001) | 0.76 (<0.001) | 0.80 (<0.001) | 0.82 (<0.001) |
|  |  | n | 150–82 | 82–84 | 84–82 | 82–84 |
|  |  | Sleep/rest fatigue | 0.40 (<0.001) | 0.66 (<0.001) | 0.67 (<0.001) | 0.83 (<0.001) |
|  |  | n | 150–82 | 82–84 | 84–82 | 82–84 |
|  |  | Cognitive Fatigue | 0.65 (<0.001) | 0.79 (<0.001) | 0.69 (<0.001) | 0.76 (<0.001) |
|  |  | n | 150–81 | 81–83 | 83–81 | 81–84 |
